# Climate change will increase the naturalization risk from garden plants in Europe

**DOI:** 10.1111/geb.12512

**Published:** 2016-08-25

**Authors:** Iwona Dullinger, Johannes Wessely, Oliver Bossdorf, Wayne Dawson, Franz Essl, Andreas Gattringer, Günther Klonner, Holger Kreft, Michael Kuttner, Dietmar Moser, Jan Pergl, Petr Pyšek, Wilfried Thuiller, Mark van Kleunen, Patrick Weigelt, Marten Winter, Stefan Dullinger, Linda Beaumont

**Affiliations:** ^1^ Division of Conservation Biology, Vegetation‐ and Landscape Ecology, Department of Botany and Biodiversity ResearchUniversity of ViennaRennweg 14Vienna1030Austria; ^2^ Institute of Social Ecology, Faculty for Interdisciplinary Studies, Alps Adria UniversitySchottenfeldgasse 29Vienna1070Austria; ^3^ Institute of Evolution and Ecology, University of TübingenAuf der Morgenstelle 5Tübingen72076Germany; ^4^ Ecology, Department of BiologyUniversity of KonstanzUniversitätsstrasse 10Konstanz78457Germany; ^5^ School of Biological and Biomedical SciencesDurham UniversitySouth RoadDurhamDH1 3LEUK; ^6^ Department of Biodiversity, Macroecology and BiogeographyUniversity of GöttingenBüsgenweg 1Göttingen37077Germany; ^7^ Department of Invasion EcologyInstitute of Botany, The Czech Academy of SciencesPrůhonice25243Czech Republic; ^8^ Department of Ecology, Faculty of ScienceCharles University in PragueViničná 7Prague12844Czech Republic; ^9^ Laboratoire d’Écologie Alpine (LECA), University of Grenoble AlpesGrenoble38000France; ^10^ Laboratoire d’Écologie Alpine (LECA), CNRSGrenoble38000France; ^11^ German Centre for Integrative Biodiversity Research (iDiv)Halle‐Jena‐Leipzig, Deutscher Platz 5eLeipzig04103Germany

**Keywords:** Alien species, horticulture, hotspot analysis, invasion debt, ornamental plants, species distribution model

## Abstract

**Aim:**

Plant invasions often follow initial introduction with a considerable delay. The current non‐native flora of a region may hence contain species that are not yet naturalized but may become so in the future, especially if climate change lifts limitations on species spread. In Europe, non‐native garden plants represent a huge pool of potential future invaders. Here, we evaluate the naturalization risk from this species pool and how it may change under a warmer climate.

**Location:**

Europe.

**Methods:**

We selected all species naturalized anywhere in the world but not yet in Europe from the set of non‐native European garden plants. For this subset of 783 species, we used species distribution models to assess their potential European ranges under different scenarios of climate change. Moreover, we defined geographical hotspots of naturalization risk from those species by combining projections of climatic suitability with maps of the area available for ornamental plant cultivation.

**Results:**

Under current climate, 165 species would already find suitable conditions in > 5% of Europe. Although climate change substantially increases the potential range of many species, there are also some that are predicted to lose climatically suitable area under a changing climate, particularly species native to boreal and Mediterranean biomes. Overall, hotspots of naturalization risk defined by climatic suitability alone, or by a combination of climatic suitability and appropriate land cover, are projected to increase by up to 102% or 64%, respectively.

**Main conclusions:**

Our results suggest that the risk of naturalization of European garden plants will increase with warming climate, and thus it is very likely that the risk of negative impacts from invasion by these plants will also grow. It is therefore crucial to increase awareness of the possibility of biological invasions among horticulturalists, particularly in the face of a warming climate.

## Introduction

Biological invasions can be conceptualized as a series of consecutive stages – from transport out of the native range to introduction into a new territory, naturalization or establishment of self‐sustaining populations, and spread across the introduced range (e.g. Blackburn *et al*., 2011). The term ‘invasive’ or ‘invader’ is thereby commonly reserved for species that have rapidly spread into multiple sites across a large area. To pass on to the next stage a species has to overcome specific barriers to its survival, establishment and spread. Whether and how fast a species manages to pass these barriers depends on a number of interacting factors that can be grouped into those relating to anthropogenic propagule pressure, physical conditions of the recipient area and biotic traits of the invader itself as well as of the invaded communites (Catford *et al*., 2009). As a result of these consecutive filters, the number of species at each stage diminishes (Williamson & Fitter, 1996), and, even for eventually succesful invaders, extensive time lags may separate first introduction, naturalization and subsequent spread (Essl *et al*., 2011).

As climatic suitability of the new territory is particularly crucial for naturalization and spread (Catford *et al*., 2009), expected climate change may importantly modify the number and identity of already introduced species able to pass to these subsequent invasion stages. Indeed, many examples have already been documented of alien species that have naturalized and/or started to spread in a region because recent warming trends have lifted former climatic limitations (Walther *et al*., 2009). Predicting which species from a given pool of non‐natives might actually benefit from upcoming climate warming, and where these species might become naturalized or invasive in the future, would provide a valuable basis for proactive management (Bradley *et al*., 2012). So far, however, research efforts have concentrated on potential range expansions of species that have already become harmful (e.g. O'Donnell *et al*., 2012; Bellard *et al*., 2013) or at least naturalized (Duursma *et al*., 2013) in the recipient area. These pre‐selections exclude potentially large numbers of species introduced but not yet naturalized or invasive, which make up the pending invasion debt of a region (Essl *et al*., 2011).

Alien species are introduced to recipient areas via different pathways (Hulme *et al*., 2008). For vascular plants, intentional introduction for ornamental use has been identified as the major pathway world‐wide (Hulme *et al*., 2008). In Europe, for example, more than 16,000 species from more than 200 families are currently in cultivation for ornamental purposes (Cullen *et al*., 2011). Public and domestic gardens thus contain the greatest pool of non‐native plants on the continent (Niinemets & Peñuelas, 2008). The chance that in a warming Europe future invaders will primarily emerge from this pool is further increased by the fact that garden plants are often cultivated beyond the climatic limits of their natural populations and hence may get ‘a head start on climate change’ (Van der Veken *et al*., 2008). In addition, horticulture often selects for traits that also promote naturalization and spread, such as rapid growth, early and prolific reproduction and disease resistance (Mack, 2000; Pemberton & Liu, 2009; Chrobock *et al*., 2011).

It remains hard to predict which particular species from the pool of introduced garden plants will actually manage to naturalize or even become invasive. What we do know, however, is: (1) species that have already managed to become naturalized somewhere in the world are more likely to escape from cultivation in other regions too (Williamson, 1999); and (2) that climate matching between native and introduced range is one of the few factors that consistently predicts invasion success across taxonomic groups and regions (Thuiller *et al*., 2005; Hayes & Barry, 2008). Using these two ‘filters’ should hence help to at least select a subset of species with a higher risk of future naturalization and spread.

Here, we follow this rationale and explore whether the naturalization risk from currently cultivated garden plants will increase under a warmer climate in Europe. In essence, we first define the pool of non‐native garden plants that have already naturalized as aliens somewhere outside of the continent, but not in Europe itself. Second, we parameterize species distribution models and use them to assess to what extent these species would already find suitable conditions for naturalization under the current climate and whether potential alien ranges would increase, on average, under three scenarios of climate warming. Third, we combine predictions for individual species into a ‘hotspot analysis’ (O'Donnell *et al*., 2012; Bellard *et al*., 2013) to identify areas with the highest numbers of potential future invaders under both current and future climatic conditions. Finally, we overlay these climatic hotspot maps with a weighted land‐cover map accounting for the amount of potential ornamental planting area of each land‐cover class (EEA, 2000) as an indicator of generic propagule pressure from gardening and urban landscaping.

## Methods

### Data

#### Species selection and data

We selected from the European Garden Flora (EGF; Cullen *et al*., 2011) all vascular plant species not native to Europe. (The EGF is the most comprehensive encyclopaedia of ornamental plants in Europe.) From this pool of species, we selected those which have successfully naturalized somewhere outside Europe but not yet anywhere in Europe, based on the Global Naturalized Alien Flora (GloNAF; van Kleunen *et al*., 2015), a newly established global alien plant species distribution database which contains lists of naturalized alien plants in more than 850 regions covering 83% of the world's terrestrial area. Cultivated taxa flagged as varieties or subspecies in the EGF were excluded to avoid overestimation when modelling the niches of the respective species. Moreover, we did not consider any taxa marked in the EGF as hybrids.

For this species subset, we then collated distribution data from the Global Biodiversity Information Facility (GBIF, http://www.gbif.org) using the rgbif library in R (Chamberlain *et al*., 2015). All species were cross‐checked for synonyms using The Plant List (http://www.theplantlist.org). Duplicates (i.e. multiple occurrences within 10′ × 10′ grid cells) and obviously erroneous records, i.e. those on an ocean surface, were removed. After these cleaning steps, we retained 783 species with more than 50 occurrences irrespective of whether these stem from the species’ native or non‐native ranges (Gallien *et al*., 2010; see Appendix S1 in Supporting Information).

#### Climate data

To characterize present‐day climate, we used climatic data (averaged for the baseline period 1950–2000) from the WorldClim database (Hijmans *et al*., 2005, www.worldclim.org) at a 10′ resolution. From the 19 bioclimatic variables provided by WorldClim, we selected six which, in combination, represent a range of regional temperature and precipitation conditions together with an estimate of seasonal variability, and which are known to influence species distributions (Root *et al*., 2003): (1) temperature seasonality, (2) maximum temperature of the warmest month, (3) minimum temperature of the coldest month, (4) precipitation seasonality, (5) precipitation of the wettest quarter and (6) precipitation of the driest quarter. Correlations (Pearson's *r*) among these variables were < 0.75 throughout and the impact of multicollinearity on model projections should hence be negligible (Dormann *et al*., 2013).

Future climate was characterized by three different IPCC5 scenarios from the new Representative Concentration Pathways family: RCP2.6 (‘mild’ scenario), RCP4.5 (‘intermediate’ scenario) and RCP8.5 (‘severe’ scenario). Based on climatic models available at the Cordex portal (http://www.euro-cordex.net), we calculated mean predicted values of the six selected bioclimatic variables for the years 2050–2100 under these three scenarios (for detailed model selection and downscaling procedure see Appendix S2).

#### Land‐cover data

For the calculation of land‐cover weighted risk maps, we used CORINE land‐cover (CLC) data at a resolution of 100 m (EEA, 2000). The CLC land‐cover classes were weighted by the estimated proportional area available for ornamental plant cultivation according to the descriptions in EEA (2000; cf. Chytrý *et al*., 2009, for a similar approach). To safeguard against rating errors, we used three different weighting schemes, i.e. three different estimates of this proportional area per land‐cover class (see Appendix S3 for details). In all three schemes, the highest weights were given to classes including private and public garden spaces (e.g. green urban areas). Within each scheme, we subsequently calculated the area‐weighted means of these proportions for each 10′ × 10′ raster cell.

### Species distribution models

#### Model parameterization and evaluation

We modelled the global realized climatic niche of each species by combining available occurrence data with current climatic data within the biomod2 platform (Thuiller *et al*., 2009) in R (R Development Core Team, 2014). The four modelling algorithms used were: generalized linear model (GLM), general additive model (GAM), boosted regression tree (BRT) and random forest (RF). Since those algorithms require presence and absence data, but GBIF provides just ‘presence‐only’ information, we generated ‘pseudo‐absences’ following the recommendations of Barbet‐Massin *et al*. (2012): for the regression technique models (GLM and GAM), we used 10,000 randomly distributed absences, and for machine‐learning technique models (BRT and RF), we used a number of pseudo‐absences equal to the number of occurrences found in GBIF and selected outside a radius of 200 km around these occurrences. For the latter approach, pseudo‐absence generation, and hence model calibration, was repeated ten times per species to ensure that selected pseudo‐absences did not bias the final predictions. For all models, the weighted sum of presences equalled the weighted sum of pseudo‐absences. The predictive performance of the models was evaluated by means of the true skill statistic (TSS; Allouche *et al*., 2006) based on a repeated (three times) split‐sampling approach in which models were calibrated with 80% of the data and evaluated over the remaining 20%.

#### Model projections

Calibrated models were used to project the climatically suitable area for each species in Europe under current and possible future climatic conditions by means of an ensemble forecast approach (Araújo & New, 2007). As pseudo‐absence generation differed between the two groups of models, we generated two separate ensemble predictions for each species, one from a combination of GLM and GAM, and one from a combination of BRT and RF models. In other words, the model projections from the repeated split‐sampling approach (and from the repeated pseudo‐absence selection in the case of BRT and RF) were aggregated to a weighted mean of projections. The contribution of each model to the ensemble forecast was weighted according to its TSS score. Models with a TSS score < 0.5 were excluded from building projections (see Appendix S4 for full information on model performance). The two probabilistic ensemble forecasts were translated into two binary maps using the value that maximizes the TSS score as the threshold for distinguishing presence and absence predictions. The two binary maps were then combined to a final consensus map where a 10′ cell was defined to be suitable for a species (under a particular climate scenario) only if both binary ensemble layers predicted its presence. The latter decision rule makes the projections conservative, i.e. the extent of climatically suitable habitat is likely to be under‐ rather than overestimated.

To assess whether potential alien ranges of the 783 species will, on average, increase, decrease or remain constant in Europe under future climates, we compared SDM projections under current and future climates in terms of the number of cells predicted to be suitable for these species. As the distribution of these numbers was highly skewed, with an excess of zeros, we used a permutation test to evaluate the significance of differences: for each species, we randomly reshuffled the number of cells predicted to occur under current conditions and the future scenario, respectively, and calculated the difference (cells in the future scenario minus cells under current conditions). This calculation was done 1000 times, resulting in a vector of 1000 mean differences among the 783 species, which is normally distributed and centred around zero. Finally, we assessed if the actually observed difference was within or outside the central 95 or 99.9% of the simulated differences.

To analyse whether possible increases or decreases of alien ranges under climate change might depend on a species’ biogeographical origin, we assigned the native regions of our study species to the nine climatically defined zonobiomes distinguished by Walter & Breckle (1991). Native regions were available for 704 of the 783 species in the GRIN database (http://www.ars-grin.gov/). Where native regions were assigned to more than one zonobiome, species were assigned to all of these zonobiomes. Finally, we re‐did the same permutation tests as described above for the subset of species of each zonobiome separately.

### Hotspot analysis and risk maps

For each climatic scenario, final binary consensus maps of all 783 species were stacked. From this overlay, we calculated for each 10′ grid cell (*c*. 220 km^2^ at latitude 50° N) the number of species that would find suitable climatic conditions there. We defined potential naturalization hotspots as the 10% of cells that provide a suitable climate to the highest numbers of species. To depict potential contraction or expansion of hotspots, we mapped the relative change in the areal extent of hotspots in comparison with the current climatic situation by applying the top 10% cut‐off value (i.e. the number of species that separates the top 10% of the grid cells from the rest) determined under current conditions to the future climatic scenarios, too.

The hotspot maps represent the number of species that are predicted to be able to naturalize in particular regions (10′ grid cells) based on their climatic requirements alone. Actual naturalization risk, however, also depends on the spatially variable amount of potential ornamental planting area. To create risk maps, we hence combined the stacked binary projections of the 783 species with each of the three weighted CORINE land‐cover maps by multiplying the number of potential invaders by the area available for ornamental plant cultivation. We again defined hotspots of naturalization risk as the 10% of cells with the highest such multiplied values. The three resulting risk maps, one per weighting scheme of land‐cover classes, were similar, but differed in some details (cf. Appendix S5). We hence created a final consensus map where hotspots of naturalization risk were defined as those cells flagged as such by at least two of the three alternative risk maps.

## Results

### Model projections and hotspot analysis

**Figure 1:**
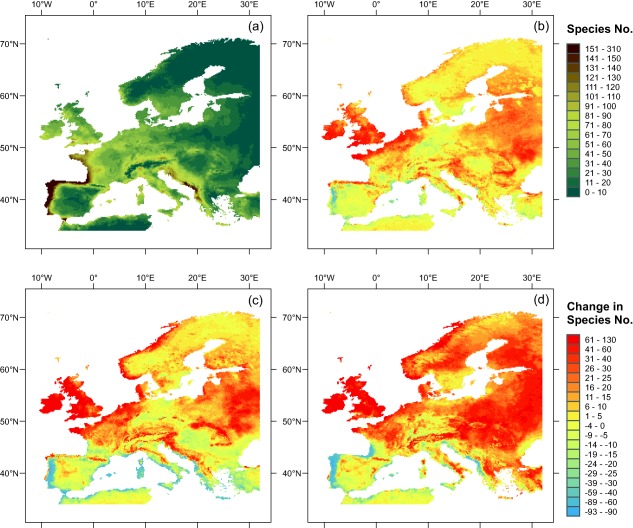
Projected climatic suitability for 783 ornamental species currently not naturalized in but somewhere outside of Europe in 10′ × 10′ grid cells. The figure shows the total numbers of species that are projected to encounter climatically suitable conditions per grid cell under current climate (a), and changes to these numbers under three different climate change scenarios (b–d).

For 455 (*c*. 58%) of the 783 species included in our analysis, there is already a certain amount of suitable habitat (> 100 cells) in Europe under current climatic conditions. The number of suitable grid cells varies considerably among species (minimum 0, maximum 18,059, i.e. *c*. 58% of Europe), but is already > 1600 cells (*c*. 5% of Europe) for 21% of the species (165 species). Per raster cell, the number of species predicted to encounter suitable climatic conditions ranges between 0 and 305 (Fig. 1a). Northern and eastern Europe currently appear least suitable and western and southern Europe most suitable for our study species.

**Figure 2:**
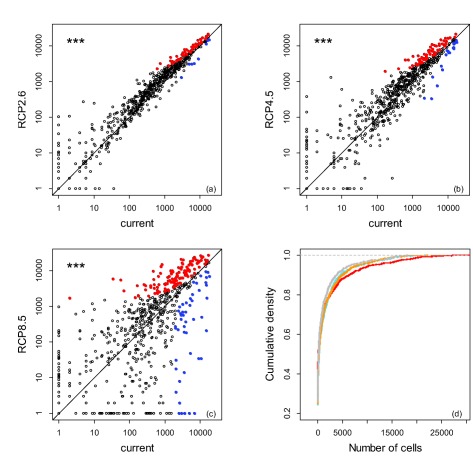
(a)–(c) Comparison of the number of cells climatically suitable for the 783 ornamental species under current climatic conditions and three different climate change scenarios (RCP2.6, RCP4.5, RCP8.5). Asterisks symbolize significant differences in the mean number of cells (*P* < 0.001). Blue and red points symbolize species that loose or gain > 1600 cells (*c*. 5% of the study area) in comparison with current climate conditions, respectively. (d) Cumulative density of the number of cells occupied by the species, i.e. the probability that a randomly selected species has a climatically suitable range < *x* under current climatic conditions (grey), and under the three climatic scenarios (RCP2.6, light blue; RCP4.5, orange; RCP 8.6, red). In (a)–(c) axes are log‐scaled.

**Figure 3:**
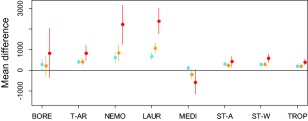
Mean difference in the number of cells climatically suitable to the 783 ornamental species under current climatic conditions and three different climate change scenarios (RCP2.6, RCP4.5, RCP8.5), separated by species zonobiome of origin. Points symbolize observed mean differences and lines 0.95 confidence intervals as derived from permutation tests. Key: blue, RCP2.6; orange, RCP4.5; red, RCP8.5; BORE, boreal; T‐AR, temperate‐arid; NEMO, nemoral (= temperate); LAUR, laurophyllous; MEDI, Mediterranean; ST‐A, subtropical‐arid; ST‐W, subtropical seasonally dry; TROP, tropical.

Under a warmer climate, both the mean potential range size per study species (Fig. 2a–c) and the number of species finding particularly large climatically suitable ranges in Europe (Fig. 2d) increase. Enlargement of mean potential range sizes is greater the more pronounced the climate‐change scenario (Fig. 3). However, not all the analysed species are predicted to profit from warmer climates. The modelled species pool is separated into those likely to gain and those which will lose climatically suitable area in a warmer Europe. The gap between these two groups becomes, again, the more pronounced the more severe the climatic scenario (Fig. 2a–c).

Separating species according to their biogeographical origin demonstrates that those native to nemoral and laurophyllous zonobiomes profit most, especially under the most severe scenario, while those native to boreal and Mediterranean zonobiomes benefit least or even decrease in mean range size under the most severe climate scenario (Fig. 3). However, at least some species from any zonobiome show particularly strong reduction or enlargement of potential range size under each climate scenario, with pronounced losers being particularly frequent among boreal, nemoral and Mediterranean species (Fig. 2, Appendix S6).

Similar to species, geographical regions are also separated into those gaining and losing potential invaders with a warming climate (Fig. 1b–d). Gains are particularly pronounced in the north‐western and eastern parts of Europe while the southern Atlantic and most of the Mediterranean coast are predicted to be suitable for a lower number of ornamentals under future climates.

**Figure 4:**
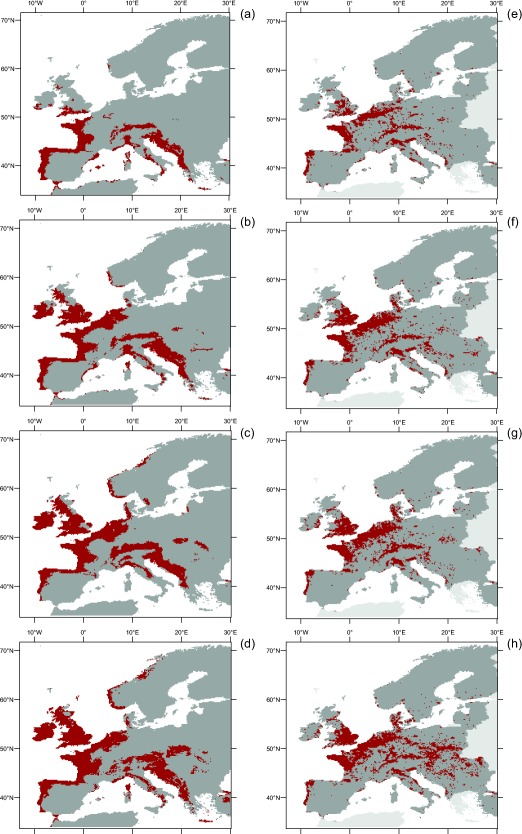
Geographical distribution of hotspots of potentially suitable climatic conditions for 783 ornamental species not yet naturalized in, but somewhere outside of Europe, under current climate (a) and three scenarios of climate warming: (b) mild scenario (RCP2.6), (c) intermediate scenario (RCP4.5) and (d) strong scenario (RCP8.5). (e)–(h) Maps of high naturalization risk calculated from combining climatic suitability under these four different assumptions of climatic conditions with the estimated area available for ornamental plant cultivation.

Under current climatic conditions 10% of Europe is climatically suitable for at least 70 from our pool of 783 species. These climatic hotspots are clustered along the Atlantic coast of Portugal, Spain, France and the southern British Isles as well as along the Mediterranean coast of the Balkan Peninsula and in southern central Europe (Fig. 4a). Under future climates, the hotspot area is predicted to grow, i.e. the area that provides climatically suitable habitat to ≥ 70 species will become larger by 62% under RCP2.6, by 75% under RCP4.5 and by 102% under RCP8.5 (i.e. more than doubling) (Fig. 4b–d).

Although part of the southern Atlantic and the Balkan coasts will lose potential invaders under climate warming (Fig. 1), they nevertheless remain among those areas climatically suitable to a particularly high proportion of the analysed ornamental plants. The increasing extent of climatic hotspot area is mainly driven by a gradual expansion to the north including most of the British Isles, parts of north‐western continental Europe, southern Norway and the western Pannonian region (Fig. 4b–d). However, most of northern and eastern Europe still does not qualify as a climatic hotspot, even under the most severe climatic scenario, although the number of potential invaders increases considerably there (Fig. 1b–d).

### Risk maps

Similar to the extent of climatic hotspots, the area of high naturalization risk is predicted to grow under climate warming by 28% under RCP2.6, by 30% under RCP4.5 and by 68% under RCP8.5 (Fig. 4f–h). Weighting by land‐cover, however, results in some important changes to the purely climatic hotspot patterns (Fig. 4e–h). High‐risk areas tend to extend further eastwards into densely populated areas of central and eastern Europe under all climate scenarios. By contrast, most of the Balkan coastal regions as well as parts of the Spanish coast are climatic hotspots under all scenarios but do not qualify as high‐risk areas. Finally, parts of north‐western Europe (e.g. Ireland, Scotland) and the southern Scandinavian coast become climatic hotspots when climate warms, but still do not appear to be areas with high naturalization risk.

## Discussion

Our results demonstrate that there is a sizeable pool of species which: (1) are planted in European gardens, at least locally, and hence already exert a certain amount of propagule pressure, (2) have proven their naturalization capacity in other parts of the world, and (3) find abundant suitable climatic space in Europe. The risk that at least some of these species will become naturalized in Europe in the future appears substantial, and it is likely that this risk will increase as climate change intensifies.

### Geographical distribution of current climatic hotspots

Climatically suitable areas for potential naturalization of garden plants are unequally distributed across Europe. Most parts of northern and eastern Europe are unsuitable for the vast majority of the analysed species under current climatic conditions, whereas hotspots are concentrated along the southern and western Atlantic shorelines and the eastern Adriatic coast. This geographical contrast suggests that not only temperature but a combination of temperature and precipitation regimes controls current patterns of climatic suitability for garden plants in Europe. The peculiarity of the Atlantic coastal areas, in particular, is a combination of relatively mild winters and humid summers keeping both frost and aridity stress low. These areas are hence likely to be within physiological tolerance limits of species from a wide array of different origins. By contrast, the Mediterranean region is warm enough in winter for nearly all selected species to be cultivated (Cullen *et al*., 2011), but arid summers represent a climatic filter to naturalization. In line with this interpretation, the Balkan coastal area, which receives more precipitation than all other parts of the Mediterranean coast in Europe, is the only Mediterranean region that ranks among potential naturalization hotspots. In the eastern and northern parts of Europe, the climate is generally colder and/or more continental, with low winter temperatures, dry summers or a combination of both. These conditions are obviously hostile to the naturalization of most species from the current pool of European garden plants.

### Effects of climate change

Release from climatic restrictions has been identified as a major potential driver of rising invasion risk under climate warming (e.g. Walther *et al*., 2009). Our results generally support this notion. The prevailing pattern detected is an increasing number of potential invaders, in particular of laurophyllous and nemoral origin, in more northern and eastern parts of Europe and a concurrent shift of potential naturalization hotspots. This predicted expansion of climatically suitable ranges is particularly worrisome in the case of ornamental plants because many of them are already cultivated far beyond conditions that would currently allow population establishment in the wild (Van der Veken *et al*., 2008). The presence of species propagules in regions that become newly climatically suitable to them effectively lifts dispersal limitations, and may therefore allow the naturalization of garden plants to keep track with climate change more closely than is commonly assumed for native plants (e.g. Corlett & Westcott, 2013).

The mean increase of climatically suitable area, however, masks pronounced variation among species. For a sizeable minority of the study species, the potential range is predicted to shrink under climate change, and under the most pronounced scenario the number of species finding suitable climate in < 1% of the European area (320 cells) is approximately the same as under current conditions (442 vs. 441 species). The reasons for climatic range loss are likely to differ among individual species, but the fact that ‘losers’ are particularly widespread among species of boreal and Mediterranean origin suggests that two factors may be of particular importance. First, species adapted to cool conditions might lose potential area because temperatures become too warm in most parts of Europe. Second, species that would currently find climatically suitable area in Mediterranean Europe may not be able to deal with the more arid conditions that are predicted for these regions (Mariotti *et al*., 2008) while, simultaneously, winter temperature does not become warm enough to compensate for such loss by expansion to the more northern, temperate parts of Europe. In accordance with the latter assumption, the regions that are currently both warm and relatively moist but will become drier in the future, like the southern Atlantic coast and the Balkan coastal area, are (1) predicted to lose the highest numbers of potential invaders and (2) are geographically separated from the more northern areas that show highest increases in the number of potential invaders.

### Combining climatic suitability and potential ornamental planting area

Urban and suburban areas usually function as centres of introduction and cultivation for ornamentals, and the proportion of introduced species usually decreases dramatically along an urban–rural gradient (Kowarik, 1995; Niinemets & Peñuelas, 2008). Combining projections of climatic suitability with the proportional area of the respective land‐use types hence pinpoints some densely populated and economically prosperous regions in Europe as potential naturalization hotspots despite a sub‐optimal climate, e.g. Great Britain under current climatic conditions. By contrast, relatively large areas appear less threatened although they would be climatically suited to many garden plants, at least under a warmer climate, like most of the coastal Balkan Peninsula, Ireland or some southern parts of coastal Scandinavia.

The risk maps presented here assume, however, that current land‐cover patterns in Europe remain unchanged. Whether and how these patterns will change depends on future European socio‐economic policies (Spangenberg *et al*., 2012). Interestingly, a recent study projecting invasion levels in Europe as dependent on land‐use change scenarios for the 21st century revealed patterns that partly resemble those found in our study, particularly with respect to rising naturalization risk in north‐western and northern Europe (Chytrý *et al*., 2012). Taken together, these parts of Europe will hence offer both climatically more suitable conditions and land‐use patterns more susceptible to alien plant establishment in the future. By contrast, in the easternmost parts of the continent rising climatic suitability to potential invaders might be attenuated by abandonment and loss of former agricultural land in these economically marginal areas (Chytrý *et al*., 2012; Spangenberg *et al*., 2012).

### Caveats

The use of species distribution models to predict range shifts under changing climatic conditions has important limitations, mainly related to the disregard of biotic interactions (e.g. Wisz *et al*., 2013), intraspecific variation in niche breadth (Valladares *et al*., 2014), dispersal limitations (Svenning & Skov, 2007) and, particularly in an invasion context, possible niche shifts (Early & Sax, 2014). In the case of our study, biotic interactions may be of limited relevance because the spatial resolution of our predictions is far beyond the scale at which plants usually interact (Pearson & Dawson, 2003). Likewise, dispersal limitation is probably less relevant as we model potential ranges of species that are actively distributed by humans, and for which the frequency of long‐distance dispersal events can be expected to rise sharply in the future with the growing importance of e‐commerce in the ornamental plant trade (Lenda *et al*., 2014; Humair *et al*., 2015). However, not all the plants modelled here will be traded and cultivated with equal intensity, and even of those planted frequently, only a subset will escape into the wild (Dehnen‐Schmutz *et al*., 2007). We hence stress that the numbers of species predicted in our study should not be taken at face value but represent a measure of spatial and temporal variation of naturalization risk. On the other hand, we note that the pool of potential invaders among European garden plants might be even larger than assumed here because species could become established or even invasive in Europe although they have not yet done so in other regions of the world. Finally, with respect to niche shifts, we took care to parameterize our models not only with data from the native ranges of the species but also from all those areas where they have already naturalized. While this strategy should characterize the climatic potential of species as accurately as possible, further changes to realized niches during their possible future establishment and invasion in Europe can of course not be completely excluded.

The reliability of species distribution models depends on the quality of the data used to fit them. GBIF combines the advantage of global coverage, and hence the possibility to fit niches of species comprehensively, with the disadvantage of the errors and biases implicit in such large databases (Meyer *et al*., 2016). However, we do not think that these errors and biases affect our results qualitatively. First, we took care to handle taxonomic problems and spatial errors when extracting occurrence data. Second, the poor coverage of northern Asia, and Russia in particular, which is probably the most important geographical bias of GBIF in our context, has little impact on our results as the number of species native to Russia in our pool is low (38 species). In addition, the detected increase of the invasion level is especially pronounced for species from nemoral and laurophyllous zonobiomes, which are mostly situated in regions with especially high record densities. Third, although predictions for individual species might suffer from inaccuracies, the multispecies patterns predicted here are consistently interpretable in terms of geographical gradients of climatic harshness in Europe, and hence appear highly plausible.

### Conclusions

One of the greatest uncertainties in assessing the invasion risk of ornamental plants comes from the difficulty of estimating the potential impacts of climate change (Dehnen‐Schmutz, 2011). Despite pronounced species‐specific differences, our results suggest that climate warming leads to an increase in currently cultivated garden plants able to naturalize in Europe as well as the area across which they may spread. Which species will eventually become invasive or have a negative environmental and/or economic impact cannot be inferred from our models. However, a larger number of naturalized species probably also implies a greater risk of impact if the ratio of naturalized and harmful species remains about constant (Jeschke & Strayer, 2005). In addition, the growing importance of trade in ornamental plants via the internet (Humair *et al*., 2015) increasingly removes any limitations on the availability of particular plants for the individual customer and hence largely eliminates the dispersal barriers that control range responses of non‐cultivated species to climate warming (Svenning & Sandel, 2013). As a corollary, raising awareness of the invasion problem among individuals and institutions involved in gardening, urban landscaping and the horticultural trade appears even more important in the face of a warming climate.

Biosketch **Iwona Dullinger** is a doctoral student with research interests in global change biology, conservation biology and social ecology. Her research mainly focuses on modelling the impacts of climate and land‐use change on species diversity.

## Supporting information

Additional supporting information may be found in the online version of this article at the publisher's web‐site:

**Appendix S1** Selected species and number of suitable cells under current and future climate.

**Appendix S2** Detailed model selection and downscaling procedure.

**Appendix S3** Selection and weighting of relevant CORINE land‐cover classes for risk map assessment.

**Appendix S4** Information on model performance.

**Appendix S5** Naturalization risk maps calculated according to three different weighting schemes.

**Appendix S6** Species predicted gain or loss of area under climate change.
