# Supplementary material for: Climate change will increase the naturalization risk from garden plants in Europe
Source: Glob Ecol Biogeogr. 2016 Aug 25;26(1):43–53. doi: 10.1111/geb.12512 (PMC5216452; doi:10.1111/geb.12512)
Supplement: Supplementary file 2 — Appendix S2 Detailed model selection and downscaling procedure. [file GEB-26-43-s002.docx]

*Global Ecology and Biogeography*

**Supporting Information**

**Climate change will increase the naturalization risk from garden plants in Europe**

Iwona Dullinger, Johannes Wessely, Oliver Bossdorf, Wayne Dawson, Franz Essl, Andreas Gattringer, Günther Klonner, Holger Kreft, Michael Kuttner, Dietmar Moser, Jan Pergl, Petr Pyšek, Wilfried Thuiller, Mark van Kleunen, Patrick Weigelt, Marten Winter, Stefan Dullinger

**Appendix S2.** Detailed model selection and downscaling procedure.
The three different scenarios (RCP2.6, RCP4.5 and RCP8.5) we used reflect different radiative forcing trajectories for the 21^st^ century relative to pre-industrial conditions: The RCP2.6 scenario assumes that radiative forcing peaks at ~ 3 W m^-2^ before 2100 and then declines and is therefore referred to as mild scenario. In the intermediate scenario, RCP4.5, radiative forcing amounts to ~ 4.5 W m^-2^ at stabilization after 2100, while in the severe scenario, RCP8.5, radiative forcing continues to rise throughout the 21^st^ century and reaches > 8.5 W m^-2^ in 2100 (Moss *et al.*, 2010). From all available models at the Cordex data portal (www.euro-cordex.net) future climate data (daily near surface temperature, monthly precipitation) were extracted. For each scenario, we then selected one model providing a relatively smooth time series of future climate parameters, namely: ICHEC-EC-EARTH_**rcp26**_r12i1p1_SMHI-RCA4, CNRM-CERFACS-CNRM-CM5_**rcp45**_r1i1p1_SMHI-RCA4, EUR-11_ICHEC-EC-EARTH_**rcp85**_r3i1p1_DMI-HIRHAM5, from now on referred to as RCP2.6, RCP4.5 and RCP8.5, respectively. Subsequent processing of these data included the following steps: (1) download of hindcast projections of the specific climate models, (2) deriving minimum, maximum and mean monthly temperatures from the daily values, (3) calculation of anomalies, i.e. temperature differences and precipitation quotients between future climate and their hindcast projections, (4) spatial interpolation of these anomalies to the 10’ resolution surface using the natural neighbour method, and (5) addition or multiplication, respectively, of the interpolated temperature and precipitation anomalies to/with the 10’ resolution current climate data from WorldClim. The resulting annual time series of future minimum, maximum and mean temperature and precipitation sums per month were averaged for the years 2050-2100 and the six bioclimatic variables selected for modelling were then recalculated from these average values. For further details on the downscaling methods see Dullinger *et al.*(2012).

REFERENCES

Dullinger, S., Gattringer, A., Thuiller, W., Moser, D., Zimmermann, N.E., Guisan, A., Willner, W., Plutzar, C., Leitner, M., Mang, T., Caccianiga, M., Dirnböck, T., Ertl, S., Fischer, A., Lenoir, J., Svenning, J.C., Psomas, A., Schmatz, D.R., Silc, U., Vittoz, P. & Hülber, K. (2012) Extinction debt of high-mountain plants under twenty-first-century climate change. *Nature Climate Change*, **2**, 619-622.

Moss, R.H., Edmonds, J.A., Hibbard, K.A., Manning, M.R., Rose, S.K., Van Vuuren, D.P., Carter, T.R., Emori, S., Kainuma, M., Kram, T., Meehl, G.A., Mitchell, J.F.B., Nakicenovic, N., Riahi, K., Smith, S.J., Stouffer, R.J., Thomson, A.M., Weyant, J.P. & Wilbanks, T.J. (2010) The next generation of scenarios for climate change research and assessment. *Nature*, **463**, 747-756.
