# Supplementary material for: Climate change will increase the naturalization risk from garden plants in Europe
Source: Glob Ecol Biogeogr. 2016 Aug 25;26(1):43–53. doi: 10.1111/geb.12512 (PMC5216452; doi:10.1111/geb.12512)
Supplement: Supplementary file 3 — Appendix S3 Selection and weighting of relevant CORINE land‐cover classes for risk map assessment. [file GEB-26-43-s003.docx]

*Global Ecology and Biogeography*

**Supporting Information**

**Climate change will increase the naturalization risk from garden plants in Europe**

Iwona Dullinger, Johannes Wessely, Oliver Bossdorf, Wayne Dawson, Franz Essl, Andreas Gattringer, Günther Klonner, Holger Kreft, Michael Kuttner, Dietmar Moser, Jan Pergl, Petr Pyšek, Wilfried Thuiller, Mark van Kleunen, Patrick Weigelt, Marten Winter, Stefan Dullinger

**Appendix S3.** Selection and weighting of relevant CORINE land-cover classes for risk map assessment.

Using the CLC class descriptions and characteristics of the class contents from the CORINE land cover technical guide ([EEA, 2000](#_ENREF_23)) we weighted CLC classes by their estimated amount of potential area for ornamental plant cultivation. As these estimates are necessarily imprecise but may have a considerable effect on the resulting risk maps, we used three separate weighting schemes that differed both in the average amount of planting area attributed to classes and in the relative weights given to each class (weighting schemes A, B, C). CLC classes with no potential ornamental area (e.g. pastures, forests and semi-natural areas) have been excluded.

Weighting scheme:

1. Artificial areas **A B C**

1.1 Urban fabric

**111 Continuous urban fabric 5 10 20**

Most of the land is covered by structures and the transport network. Building, roads and artificially surfaced areas cover more than 80 % of the total surface. Non-linear areas of vegetation and bare soil are exceptional. Includes greenery (parks and grass areas) and small cemeteries <25ha.

**112 Discontinuous urban fabric 10 15 20**

Most of the land is covered by structures. Buildings, roads and artificially surfaced areas associated with vegetated areas and bare soil, which occupy discontinuous but significant surfaces.
Includes parks, private gardens in suburbs, green spaces between blocks of flats, cemeteries <25ha, playgrounds.

1.2 Industrial, commercial and transport units

**121 Industrial or commercial unit 0 1 5**

Artificially surfaced areas (with concrete, asphalt, tarmacadam, or stabilised, e.g. beaten earth) without vegetation occupy most of the area, which also contains buildings and/or vegetation. Including stud farms, agricultural facilities (state farm centres).

**122 road and rail networks 2 3 5**

Motorways and railways, including associated installations (stations,
 platforms, embankments). Minimum width for inclusion: 100 m.
 Including linear greenery.

1.4 Artificial non-agricultural vegetated areas

**141 Green urban areas 30 75 100**

Areas with vegetation within urban fabric, includes parks and cemeteries with vegetation, and mansions and their grounds.

**142 Sport and leisure facilities 2 5 20**

Camping grounds, sports grounds, leisure parks, golf courses, racecourses, etc. Includes formal parks not surrounded by urban areas and cemeteries with vegetation situated outside of settlements, zoological and botanical gardens located outside of settlements, places of worship: e.g., convents, monasteries.

2. Agricultural areas

2.1 Arable land

**211 Non-irrigated arable land 1 2 10**

Cereals, legumes, fodder crops, root crops and fallow land. Includes flowers and fruit trees (nurseries cultivation) and vegetables, whether open field, under plastic or glass (includes market gardening). Includes aromatic, medicinal and culinary plants, nurseries cultivation/gardens, and market gardening.

2.2 Permanent crops

**222 Fruit trees and berry plantations 0 1 5**

Parcels planted with fruit trees or shrubs: single or mixed fruit species, fruit trees associated with permanently grassed surfaces, includes chestnut and walnut groves and plantations of Rosaceae.

2.4 Heterogeneous agricultural areas

**242 Complex cultivation patterns 1 2 10**

Juxtaposition of small parcels of diverse annual crops, pastures and/or
 permanent crops, including hobby city gardens.

**243 Land occupied by agriculture, with significant natural vegetation
 1 2 5**

Areas principally occupied by agriculture, interspersed with significant
natural areas, includes sporadically occurring houses of rural settlements or farm buildings and their gardens.
