# Supplementary material for: Climate change will increase the naturalization risk from garden plants in Europe
Source: Glob Ecol Biogeogr. 2016 Aug 25;26(1):43–53. doi: 10.1111/geb.12512 (PMC5216452; doi:10.1111/geb.12512)
Supplement: Supplementary file 4 — Appendix S4 Information on model performance. [file GEB-26-43-s004.docx]

*Global Ecology and Biogeography*

**Supporting Information**

**Climate change will increase the naturalization risk from garden plants in Europe**

Iwona Dullinger, Johannes Wessely, Oliver Bossdorf, Wayne Dawson, Franz Essl, Andreas Gattringer, Günther Klonner, Holger Kreft, Michael Kuttner, Dietmar Moser, Jan Pergl, Petr Pyšek, Wilfried Thuiller, Mark van Kleunen, Patrick Weigelt, Marten Winter, Stefan Dullinger

**Appendix S4.** Information on model performance.

The table lists for all species and each modelling technique the mean TSS over all replicates and the percentage of replicates with TSS < 0.5.

| **model**  **species** | **GLM** | | **GAM** | | **RF** | | **GBM** | |
| --- | --- | --- | --- | --- | --- | --- | --- | --- |
|  | **Mean TSS** | **% TSS < 0.5** | **Mean TSS** | **% TSS < 0.5** | **Mean TSS** | **% TSS < 0.5** | **Mean TSS** | **% TSS < 0.5** |
| Abelmoschus moschatus | 0.823 | 0 | 0.823 | 0 | 0.856 | 0 | 0.847 | 0 |
| Abrus precatorius | 0.823 | 0 | 0.829 | 0 | 0.876 | 0 | 0.863 | 0 |
| Acacia pravissima | 0.968 | 0 | 0.934 | 0 | 0.943 | 0 | 0.941 | 0 |
| Acalypha hispida | 0.736 | 0 | 0.708 | 0 | 0.804 | 0 | 0.802 | 0 |
| Acalypha wilkesiana | 0.759 | 0 | 0.689 | 0 | 0.809 | 0 | 0.794 | 0 |
| Acanthocereus tetragonus | 0.895 | 0 | 0.888 | 0 | 0.916 | 0 | 0.917 | 0 |
| Acer buergerianum | 0.827 | 0 | 0.783 | 0 | 0.891 | 0 | 0.864 | 3.3 |
| Acer japonicum | 0.871 | 0 | 0.937 | 0 | 0.922 | 0 | 0.902 | 0 |
| Acer palmatum | 0.884 | 0 | 0.881 | 0 | 0.921 | 0 | 0.909 | 0 |
| Acer spicatum | 0.925 | 0 | 0.938 | 0 | 0.962 | 0 | 0.959 | 0 |
| Actinidia arguta | 0.904 | 0 | 0.858 | 0 | 0.945 | 0 | 0.944 | 0 |
| Actinidia polygama | 0.96 | 0 | 0.907 | 0 | 0.947 | 0 | 0.948 | 0 |
| Adansonia digitata | 0.808 | 0 | 0.761 | 0 | 0.825 | 0 | 0.824 | 0 |
| Adenanthera pavonina | 0.813 | 0 | 0.758 | 0 | 0.876 | 0 | 0.868 | 0 |
| Adenium obesum | 0.841 | 0 | 0.805 | 0 | 0.844 | 0 | 0.846 | 0 |
| Aesculus glabra | 0.911 | 0 | 0.888 | 0 | 0.931 | 0 | 0.922 | 0 |
| Agastache rugosa | 0.864 | 0 | 0.803 | 0 | 0.862 | 0 | 0.848 | 0 |
| Ageratina ligustrina | 0.907 | 0 | 0.91 | 0 | 0.923 | 0 | 0.917 | 0 |
| Ageratum conyzoides | 0.759 | 0 | 0.761 | 0 | 0.874 | 0 | 0.852 | 0 |
| Albizia chinensis | 0.878 | 0 | 0.823 | 0 | 0.87 | 0 | 0.87 | 0 |
| Albizia lebbeck | 0.723 | 0 | 0.75 | 0 | 0.793 | 0 | 0.787 | 0 |
| Albizia saman | 0.807 | 0 | 0.796 | 0 | 0.852 | 0 | 0.85 | 0 |
| Albuca bracteata | 0.749 | 0 | 0.898 | 0 | 0.906 | 0 | 0.922 | 0 |
| Albuca canadensis | 0.937 | 0 | 0.873 | 33.3 | 0.972 | 0 | 0.956 | 0 |
| Aleurites moluccana | 0.849 | 0 | 0.81 | 0 | 0.848 | 0 | 0.848 | 0 |
| Allamanda cathartica | 0.823 | 0 | 0.828 | 0 | 0.873 | 0 | 0.871 | 0 |
| Allium tuberosum | 0.765 | 0 | 0.707 | 0 | 0.807 | 0 | 0.795 | 0 |
| Allocasuarina littoralis | 0.967 | 0 | 0.979 | 0 | 0.98 | 0 | 0.973 | 0 |
| Allocasuarina verticillata | 0.964 | 0 | 0.969 | 0 | 0.974 | 0 | 0.97 | 0 |
| Alnus nepalensis | 0.94 | 0 | 0.929 | 0 | 0.952 | 0 | 0.946 | 0 |
| Alocasia macrorrhizos | 0.835 | 0 | 0.837 | 0 | 0.861 | 0 | 0.847 | 0 |
| Alstonia scholaris | 0.882 | 0 | 0.785 | 0 | 0.842 | 0 | 0.844 | 0 |
| Alternanthera ficoidea | 0.754 | 0 | 0.815 | 0 | 0.837 | 0 | 0.83 | 0 |
| Ammobium alatum | 0.845 | 0 | 0.831 | 0 | 0.944 | 0 | 0.94 | 0 |
| Ampelopsis glandulosa | 0.9 | 0 | 0.86 | 0 | 0.944 | 0 | 0.937 | 0 |
| Anacardium occidentale | 0.811 | 0 | 0.818 | 0 | 0.889 | 0 | 0.885 | 0 |
| Ananas comosus | 0.717 | 0 | 0.73 | 0 | 0.802 | 0 | 0.781 | 0 |
| Angelonia angustifolia | 0.839 | 0 | 0.774 | 0 | 0.882 | 0 | 0.885 | 0 |
| Anigozanthos flavidus | 0.807 | 0 | 0.907 | 0 | 0.955 | 0 | 0.942 | 0 |
| Annona cherimola | 0.786 | 0 | 0.834 | 0 | 0.866 | 0 | 0.852 | 0 |
| Annona glabra | 0.845 | 0 | 0.872 | 0 | 0.885 | 0 | 0.878 | 0 |
| Annona muricata | 0.823 | 0 | 0.827 | 0 | 0.861 | 0 | 0.855 | 0 |
| Annona reticulata | 0.812 | 0 | 0.826 | 0 | 0.879 | 0 | 0.873 | 0 |
| Annona squamosa | 0.807 | 0 | 0.799 | 0 | 0.822 | 0 | 0.813 | 0 |
| Anoda cristata | 0.677 | 0 | 0.75 | 0 | 0.845 | 0 | 0.794 | 0 |
| Anthurium pentaphyllum | 0.887 | 0 | 0.873 | 0 | 0.914 | 0 | 0.915 | 0 |
| Anthurium schlechtendalii | 0.885 | 0 | 0.924 | 0 | 0.924 | 0 | 0.923 | 0 |
| Antigonon leptopus | 0.733 | 0 | 0.75 | 0 | 0.816 | 0 | 0.799 | 0 |
| Apodytes dimidiata | 0.858 | 0 | 0.883 | 0 | 0.925 | 0 | 0.907 | 0 |
| Archontophoenix cunninghamiana | 0.94 | 0 | 0.912 | 0 | 0.967 | 0 | 0.963 | 0 |
| Arctotis fastuosa | 0.884 | 0 | 0.88 | 0 | 0.944 | 0 | 0.944 | 0 |
| Ardisia crenata | 0.882 | 0 | 0.873 | 0 | 0.897 | 0 | 0.894 | 0 |
| Ardisia japonica | 0.965 | 0 | 0.953 | 0 | 0.971 | 0 | 0.967 | 0 |
| Aristolochia grandiflora | 0.84 | 0 | 0.819 | 0 | 0.856 | 0 | 0.854 | 0 |
| Artemisia ludoviciana | 0.778 | 0 | 0.81 | 0 | 0.905 | 0 | 0.842 | 0 |
| Artocarpus altilis | 0.849 | 0 | 0.829 | 0 | 0.872 | 0 | 0.868 | 0 |
| Artocarpus heterophyllus | 0.767 | 0 | 0.784 | 0 | 0.854 | 0 | 0.836 | 0 |
| Arum palaestinum | 0.874 | 0 | 0.846 | 0 | 0.945 | 0 | 0.93 | 0 |
| Arundina graminifolia | 0.856 | 0 | 0.897 | 0 | 0.892 | 0 | 0.887 | 0 |
| Asimina triloba | 0.949 | 0 | 0.956 | 0 | 0.963 | 0 | 0.954 | 0 |
| Asparagus africanus | 0.816 | 0 | 0.817 | 0 | 0.862 | 0 | 0.848 | 0 |
| Asparagus declinatus | 0.937 | 0 | 0.89 | 0 | 0.937 | 0 | 0.92 | 0 |
| Asparagus falcatus | 0.826 | 0 | 0.846 | 0 | 0.872 | 0 | 0.854 | 0 |
| Asparagus retrofractus | 0.947 | 0 | 0.96 | 0 | 0.959 | 0 | 0.951 | 0 |
| Asparagus scandens | 0.977 | 0 | 0.9 | 0 | 0.964 | 0 | 0.958 | 0 |
| Asparagus virgatus | 0.917 | 0 | 0.913 | 33.3 | 0.954 | 0 | 0.936 | 0 |
| Atriplex canescens | 0.78 | 0 | 0.805 | 0 | 0.917 | 0 | 0.872 | 0 |
| Atriplex nummularia | 0.908 | 0 | 0.91 | 0 | 0.958 | 0 | 0.952 | 0 |
| Averrhoa carambola | 0.831 | 0 | 0.754 | 0 | 0.791 | 0 | 0.794 | 0 |
| Banisteriopsis caapi | 0.801 | 0 | 0.708 | 0 | 0.912 | 0 | 0.906 | 0 |
| Banksia ericifolia | 0.961 | 0 | 0.935 | 0 | 0.987 | 0 | 0.975 | 0 |
| Barleria cristata | 0.673 | 0 | 0.618 | 0 | 0.769 | 6.7 | 0.774 | 3.3 |
| Barringtonia asiatica | 0.914 | 0 | 0.824 | 0 | 0.918 | 0 | 0.909 | 0 |
| Bartlettina sordida | 0.791 | 0 | 0.753 | 0 | 0.857 | 0 | 0.842 | 0 |
| Basella alba | 0.72 | 0 | 0.766 | 0 | 0.809 | 0 | 0.805 | 0 |
| Bauhinia galpinii | 0.894 | 0 | 0.865 | 0 | 0.919 | 0 | 0.907 | 0 |
| Bauhinia purpurea | 0.741 | 0 | 0.678 | 0 | 0.815 | 0 | 0.81 | 0 |
| Bauhinia variegata | 0.748 | 0 | 0.763 | 0 | 0.77 | 0 | 0.773 | 0 |
| Begonia cucullata | 0.782 | 0 | 0.733 | 0 | 0.858 | 0 | 0.848 | 0 |
| Begonia heracleifolia | 0.883 | 0 | 0.844 | 0 | 0.927 | 0 | 0.911 | 0 |
| Begonia humilis | 0.847 | 0 | 0.873 | 0 | 0.902 | 0 | 0.895 | 0 |
| Begonia nelumbiifolia | 0.844 | 0 | 0.786 | 0 | 0.879 | 0 | 0.875 | 0 |
| Berberis glaucocarpa | 0.92 | 0 | 0.801 | 0 | 0.961 | 0 | 0.942 | 0 |
| Berberis repens | 0.916 | 0 | 0.922 | 0 | 0.947 | 0 | 0.943 | 0 |
| Bertholletia excelsa | 0.942 | 0 | 0.741 | 33.3 | 0.925 | 0 | 0.923 | 0 |
| Betula platyphylla | NaN | 100 | 0.851 | 0 | 0.881 | 0 | 0.873 | 0 |
| Bignonia capreolata | 0.939 | 0 | 0.917 | 0 | 0.95 | 0 | 0.945 | 0 |
| Billardiera heterophylla | 0.866 | 0 | 0.886 | 0 | 0.89 | 0 | 0.883 | 0 |
| Bixa orellana | 0.807 | 0 | 0.801 | 0 | 0.881 | 0 | 0.877 | 0 |
| Bocconia frutescens | 0.818 | 0 | 0.816 | 0 | 0.913 | 0 | 0.905 | 0 |
| Boltonia asteroides | 0.849 | 0 | 0.862 | 0 | 0.929 | 0 | 0.914 | 0 |
| Bomarea multiflora | 0.957 | 0 | 0.936 | 0 | 0.966 | 0 | 0.97 | 0 |
| Bombax ceiba | 0.854 | 0 | 0.889 | 0 | 0.909 | 0 | 0.892 | 0 |
| Bouteloua curtipendula | 0.753 | 0 | 0.792 | 0 | 0.904 | 0 | 0.847 | 0 |
| Brachychiton acerifolius | 0.95 | 0 | 0.946 | 0 | 0.932 | 0 | 0.929 | 0 |
| Brachychiton discolor | 0.771 | 0 | 0.871 | 0 | 0.872 | 0 | 0.871 | 0 |
| Brachychiton populneus | 0.965 | 0 | 0.963 | 0 | 0.975 | 0 | 0.97 | 0 |
| Brexia madagascariensis | 0.916 | 0 | 0.916 | 0 | 0.942 | 0 | 0.944 | 0 |
| Breynia disticha | 0.77 | 0 | 0.791 | 0 | 0.86 | 0 | 0.857 | 0 |
| Bromus briziformis | 0.954 | 0 | 0.936 | 0 | 0.925 | 0 | 0.917 | 0 |
| Bromus danthoniae | 0.845 | 0 | 0.91 | 0 | 0.885 | 0 | 0.876 | 0 |
| Browallia americana | 0.83 | 0 | 0.871 | 0 | 0.909 | 0 | 0.903 | 0 |
| Brownea coccinea | 0.933 | 0 | 0.898 | 0 | 0.933 | 0 | 0.915 | 0 |
| Brownea grandiceps | 0.88 | 0 | 0.821 | 0 | 0.944 | 0 | 0.936 | 0 |
| Brugmansia sanguinea | 0.884 | 0 | 0.846 | 0 | 0.963 | 0 | 0.95 | 0 |
| Brunfelsia uniflora | 0.721 | 0 | 0.791 | 0 | 0.847 | 0 | 0.822 | 0 |
| Bucida buceras | 0.875 | 0 | 0.885 | 0 | 0.928 | 0 | 0.922 | 0 |
| Buddleja asiatica | 0.823 | 0 | 0.817 | 0 | 0.842 | 0 | 0.836 | 0 |
| Buddleja indica | 0.968 | 0 | 0.945 | 0 | 0.972 | 0 | 0.96 | 0 |
| Buddleja saligna | 0.95 | 0 | 0.941 | 0 | 0.942 | 0 | 0.927 | 0 |
| Buddleja salviifolia | 0.937 | 0 | 0.939 | 0 | 0.952 | 0 | 0.937 | 0 |
| Buddleja stachyoides | 0.744 | 0 | 0.821 | 0 | 0.936 | 0 | 0.904 | 0 |
| Bulbine semibarbata | 0.921 | 0 | 0.924 | 0 | 0.961 | 0 | 0.953 | 0 |
| Bursera simaruba | 0.813 | 0 | 0.826 | 0 | 0.863 | 0 | 0.846 | 0 |
| Caesalpinia coriaria | 0.84 | 0 | 0.869 | 0 | 0.871 | 0 | 0.87 | 0 |
| Caesalpinia pulcherrima | 0.691 | 0 | 0.723 | 0 | 0.816 | 0 | 0.801 | 0 |
| Caladium bicolor | 0.776 | 0 | 0.822 | 0 | 0.872 | 0 | 0.867 | 0 |
| Calceolaria chelidonioides | 0.866 | 0 | 0.814 | 0 | 0.902 | 0 | 0.909 | 0 |
| Calliandra haematocephala | 0.798 | 0 | 0.78 | 0 | 0.82 | 0 | 0.798 | 0 |
| Calliandra houstoniana | 0.838 | 0 | 0.889 | 0 | 0.91 | 0 | 0.891 | 0 |
| Calliandra surinamensis | 0.842 | 0 | 0.808 | 0 | 0.9 | 0 | 0.903 | 0 |
| Callicarpa dichotoma | 0.902 | 0 | 0.828 | 0 | 0.955 | 0 | 0.93 | 0 |
| Callicarpa japonica | 0.959 | 0 | 0.939 | 0 | 0.96 | 0 | 0.956 | 0 |
| Callisia repens | 0.771 | 0 | 0.783 | 0 | 0.837 | 0 | 0.825 | 0 |
| Callistemon speciosus | 0.756 | 0 | 0.816 | 0 | 0.861 | 0 | 0.831 | 0 |
| Callistemon viminalis | 0.818 | 0 | 0.856 | 0 | 0.9 | 0 | 0.886 | 0 |
| Calophyllum inophyllum | 0.93 | 0 | 0.932 | 0 | 0.912 | 0 | 0.91 | 0 |
| Calotropis procera | 0.699 | 0 | 0.746 | 0 | 0.807 | 0 | 0.772 | 0 |
| Calystegia hederacea | 0.935 | 0 | 0.918 | 0 | 0.974 | 0 | 0.969 | 0 |
| Camellia japonica | 0.895 | 0 | 0.912 | 0 | 0.913 | 0 | 0.912 | 0 |
| Camellia sinensis | 0.916 | 0 | 0.856 | 0 | 0.922 | 0 | 0.914 | 0 |
| Campanula punctata | 0.901 | 0 | 0.898 | 0 | 0.946 | 0 | 0.94 | 0 |
| Cananga odorata | 0.875 | 0 | 0.863 | 0 | 0.864 | 0 | 0.857 | 0 |
| Canavalia cathartica | 0.805 | 0 | 0.76 | 0 | 0.837 | 0 | 0.823 | 0 |
| Canavalia ensiformis | 0.762 | 0 | 0.713 | 0 | 0.75 | 0 | 0.75 | 0 |
| Canna glauca | 0.706 | 0 | 0.697 | 0 | 0.813 | 0 | 0.802 | 0 |
| Cardiocrinum cordatum | 0.977 | 0 | 0.96 | 0 | 0.996 | 0 | 0.991 | 0 |
| Carica papaya | 0.758 | 0 | 0.768 | 0 | 0.832 | 0 | 0.821 | 0 |
| Carissa macrocarpa | 0.649 | 0 | 0.596 | 33.3 | 0.813 | 0 | 0.773 | 0 |
| Carpinus caroliniana | 0.843 | 0 | 0.87 | 0 | 0.932 | 0 | 0.916 | 0 |
| Carya illinoinensis | 0.849 | 0 | 0.851 | 0 | 0.87 | 0 | 0.859 | 0 |
| Cascabela thevetia | 0.8 | 0 | 0.727 | 0 | 0.773 | 3.3 | 0.73 | 3.3 |
| Cassia fistula | 0.725 | 0 | 0.704 | 0 | 0.801 | 0 | 0.792 | 0 |
| Cassia grandis | 0.801 | 0 | 0.807 | 0 | 0.878 | 0 | 0.876 | 0 |
| Castanea mollissima | 0.885 | 0 | 0.835 | 0 | 0.923 | 0 | 0.906 | 0 |
| Castanospermum australe | 0.954 | 0 | 0.903 | 0 | 0.938 | 0 | 0.929 | 0 |
| Casuarina cunninghamiana | 0.853 | 0 | 0.854 | 0 | 0.922 | 0 | 0.897 | 0 |
| Casuarina glauca | 0.967 | 0 | 0.934 | 0 | 0.976 | 0 | 0.975 | 0 |
| Cedrela odorata | 0.8 | 0 | 0.8 | 0 | 0.881 | 0 | 0.869 | 0 |
| Ceiba pentandra | 0.782 | 0 | 0.792 | 0 | 0.857 | 0 | 0.854 | 0 |
| Celastrus scandens | 0.912 | 0 | 0.926 | 0 | 0.968 | 0 | 0.957 | 0 |
| Centrosema virginianum | 0.735 | 0 | 0.769 | 0 | 0.859 | 0 | 0.84 | 0 |
| Ceratopetalum gummiferum | 0.985 | 0 | 0.985 | 0 | 0.994 | 0 | 0.986 | 0 |
| Ceratotheca triloba | NaN | 100 | 0.933 | 0 | 0.976 | 0 | 0.957 | 0 |
| Cercestis mirabilis | 0.931 | 0 | 0.932 | 0 | 0.951 | 0 | 0.945 | 0 |
| Cercidiphyllum japonicum | 0.859 | 0 | 0.85 | 0 | 0.912 | 0 | 0.908 | 0 |
| Cestrum aurantiacum | 0.81 | 0 | 0.763 | 0 | 0.875 | 0 | 0.865 | 0 |
| Cestrum fasciculatum | 0.742 | 33.3 | 0.825 | 0 | 0.905 | 0 | 0.887 | 0 |
| Cestrum nocturnum | 0.819 | 0 | 0.821 | 0 | 0.863 | 0 | 0.844 | 0 |
| Chamaedorea elegans | 0.879 | 0 | 0.781 | 0 | 0.92 | 0 | 0.907 | 0 |
| Chamelaucium uncinatum | 0.808 | 0 | 0.917 | 0 | 0.953 | 0 | 0.931 | 0 |
| Cheilocostus speciosus | 0.837 | 0 | 0.845 | 0 | 0.877 | 0 | 0.879 | 0 |
| Chenopodium quinoa | 0.837 | 0 | 0.77 | 0 | 0.875 | 0 | 0.858 | 0 |
| Chrysobalanus icaco | 0.844 | 0 | 0.84 | 0 | 0.9 | 0 | 0.891 | 0 |
| Chrysophyllum cainito | 0.845 | 0 | 0.826 | 0 | 0.852 | 0 | 0.84 | 0 |
| Chrysophyllum oliviforme | 0.859 | 0 | 0.932 | 0 | 0.915 | 0 | 0.892 | 0 |
| Chrysothemis pulchella | 0.91 | 0 | 0.886 | 0 | 0.924 | 0 | 0.924 | 0 |
| Cinnamomum camphora | 0.871 | 0 | 0.862 | 0 | 0.905 | 0 | 0.902 | 0 |
| Cissus alata | 0.878 | 0 | 0.729 | 0 | 0.91 | 0 | 0.914 | 0 |
| Cissus antarctica | 0.995 | 0 | 0.985 | 0 | 0.994 | 0 | 0.991 | 0 |
| Cissus quadrangularis | 0.862 | 0 | 0.859 | 0 | 0.863 | 0 | 0.863 | 0 |
| Cissus rotundifolia | 0.884 | 0 | 0.868 | 0 | 0.886 | 0 | 0.884 | 0 |
| Cissus verticillata | 0.785 | 0 | 0.773 | 0 | 0.89 | 0 | 0.868 | 0 |
| Citharexylum spinosum | 0.863 | 0 | 0.817 | 0 | 0.898 | 0 | 0.89 | 0 |
| Citrus aurantiifolia | 0.793 | 0 | 0.772 | 0 | 0.817 | 0 | 0.812 | 0 |
| Citrus maxima | 0.717 | 0 | 0.727 | 0 | 0.811 | 0 | 0.797 | 0 |
| Clarkia amoena | 0.856 | 0 | 0.898 | 0 | 0.942 | 0 | 0.927 | 0 |
| Clarkia pulchella | 0.886 | 0 | 0.893 | 0 | 0.925 | 0 | 0.915 | 0 |
| Clematis paniculata | 0.966 | 0 | 0.945 | 0 | 0.973 | 0 | 0.964 | 0 |
| Clematis tangutica | 0.84 | 0 | 0.848 | 0 | 0.925 | 0 | 0.914 | 0 |
| Clematis terniflora | 0.832 | 0 | 0.898 | 0 | 0.928 | 0 | 0.915 | 0 |
| Cleome gynandra | 0.704 | 0 | 0.729 | 0 | 0.797 | 0 | 0.772 | 0 |
| Clerodendrum bungei | 0.918 | 0 | 0.837 | 0 | 0.833 | 0 | 0.813 | 0 |
| Clerodendrum chinense | 0.809 | 0 | 0.79 | 0 | 0.847 | 0 | 0.835 | 0 |
| Clerodendrum splendens | 0.897 | 0 | 0.887 | 0 | 0.917 | 0 | 0.911 | 0 |
| Clerodendrum trichotomum | 0.921 | 0 | 0.905 | 0 | 0.934 | 0 | 0.926 | 0 |
| Cleyera japonica | 0.952 | 0 | 0.952 | 0 | 0.957 | 0 | 0.948 | 0 |
| Clidemia hirta | 0.857 | 0 | 0.853 | 0 | 0.925 | 0 | 0.919 | 0 |
| Clitoria ternatea | 0.74 | 0 | 0.739 | 0 | 0.832 | 0 | 0.823 | 0 |
| Clusia rosea | 0.837 | 0 | 0.844 | 0 | 0.881 | 0 | 0.881 | 0 |
| Cobaea scandens | 0.875 | 0 | 0.792 | 0 | 0.917 | 0 | 0.912 | 0 |
| Coccinia grandis | 0.718 | 0 | 0.719 | 0 | 0.737 | 0 | 0.739 | 0 |
| Coccoloba uvifera | 0.835 | 0 | 0.879 | 0 | 0.857 | 0 | 0.856 | 0 |
| Cochlospermum vitifolium | 0.82 | 0 | 0.819 | 0 | 0.862 | 0 | 0.856 | 0 |
| Cocos nucifera | 0.817 | 0 | 0.806 | 0 | 0.814 | 0 | 0.819 | 0 |
| Codiaeum variegatum | 0.848 | 0 | 0.831 | 0 | 0.891 | 0 | 0.881 | 0 |
| Coffea liberica | 0.86 | 0 | 0.831 | 0 | 0.91 | 0 | 0.903 | 0 |
| Cola acuminata | 0.917 | 0 | 0.853 | 0 | 0.867 | 0 | 0.863 | 0 |
| Combretum grandiflorum | 0.952 | 0 | 0.915 | 0 | 0.909 | 0 | 0.9 | 0 |
| Combretum indicum | 0.757 | 0 | 0.747 | 0 | 0.785 | 0 | 0.782 | 0 |
| Commelina benghalensis | 0.669 | 0 | 0.712 | 0 | 0.806 | 0 | 0.768 | 0 |
| Commelina coelestis | 0.887 | 0 | 0.846 | 0 | 0.905 | 0 | 0.9 | 0 |
| Conicosia pugioniformis | 0.988 | 0 | 0.969 | 0 | 0.968 | 0 | 0.951 | 0 |
| Coprosma robusta | 0.981 | 0 | 0.941 | 0 | 0.993 | 0 | 0.987 | 0 |
| Corchorus olitorius | 0.741 | 0 | 0.746 | 0 | 0.798 | 0 | 0.78 | 0 |
| Cordyline fruticosa | 0.807 | 0 | 0.732 | 0 | 0.839 | 0 | 0.844 | 0 |
| Cortaderia jubata | 0.86 | 0 | 0.823 | 0 | 0.918 | 0 | 0.907 | 0 |
| Corylus heterophylla | 0.766 | 0 | 0.783 | 0 | 0.903 | 0 | 0.892 | 0 |
| Corymbia citriodora | 0.858 | 0 | 0.863 | 0 | 0.895 | 0 | 0.875 | 0 |
| Cosmos sulphureus | 0.683 | 0 | 0.701 | 0 | 0.791 | 0 | 0.771 | 0 |
| Cotoneaster glaucophyllus | 0.942 | 0 | 0.925 | 0 | 0.968 | 0 | 0.956 | 0 |
| Couroupita guianensis | 0.904 | 0 | 0.859 | 0 | 0.913 | 0 | 0.926 | 0 |
| Crassula ericoides | 0.968 | 0 | 0.886 | 0 | 0.961 | 0 | 0.95 | 0 |
| Crataegus pubescens | 0.935 | 0 | 0.919 | 0 | 0.944 | 0 | 0.937 | 0 |
| Crescentia cujete | 0.803 | 0 | 0.81 | 0 | 0.853 | 0 | 0.849 | 0 |
| Crinum asiaticum | 0.872 | 0 | 0.853 | 0 | 0.869 | 0 | 0.873 | 0 |
| Crinum zeylanicum | 0.864 | 0 | 0.837 | 0 | 0.853 | 0 | 0.856 | 0 |
| Crotalaria capensis | 0.944 | 0 | 0.921 | 0 | 0.952 | 0 | 0.942 | 0 |
| Crotalaria juncea | 0.738 | 0 | 0.754 | 0 | 0.803 | 0 | 0.786 | 0 |
| Crotalaria micans | 0.778 | 0 | 0.791 | 0 | 0.873 | 0 | 0.859 | 0 |
| Crotalaria retusa | 0.78 | 0 | 0.793 | 0 | 0.868 | 0 | 0.864 | 0 |
| Cucumis anguria | 0.775 | 0 | 0.793 | 0 | 0.823 | 0 | 0.811 | 0 |
| Cucumis dipsaceus | 0.706 | 0 | 0.664 | 0 | 0.82 | 0 | 0.833 | 0 |
| Cucumis metuliferus | 0.703 | 0 | 0.781 | 0 | 0.784 | 0 | 0.779 | 0 |
| Cucurbita argyrosperma | 0.795 | 0 | 0.811 | 0 | 0.839 | 0 | 0.829 | 0 |
| Cucurbita ficifolia | 0.778 | 0 | 0.777 | 0 | 0.802 | 0 | 0.793 | 0 |
| Cucurbita moschata | 0.648 | 0 | 0.677 | 0 | 0.766 | 0 | 0.733 | 0 |
| Cuphea hyssopifolia | 0.79 | 0 | 0.811 | 0 | 0.83 | 0 | 0.815 | 0 |
| Curcuma longa | 0.881 | 0 | 0.792 | 0 | 0.844 | 0 | 0.858 | 0 |
| Cymbopogon nardus | 0.892 | 0 | 0.883 | 0 | 0.906 | 0 | 0.9 | 0 |
| Cynoglossum amabile | 0.799 | 0 | 0.838 | 0 | 0.821 | 0 | 0.806 | 0 |
| Cynoglossum zeylanicum | 0.871 | 0 | 0.852 | 0 | 0.878 | 0 | 0.872 | 0 |
| Cyperus albostriatus | 0.921 | 0 | 0.913 | 0 | 0.946 | 0 | 0.933 | 0 |
| Cytisus proliferus | 0.901 | 0 | 0.909 | 0 | 0.942 | 0 | 0.935 | 0 |
| Dahlia imperialis | 0.912 | 0 | 0.917 | 0 | 0.923 | 0 | 0.92 | 0 |
| Dahlia pinnata | 0.814 | 0 | 0.858 | 0 | 0.886 | 0 | 0.874 | 0 |
| Datura ceratocaula | 0.925 | 0 | 0.88 | 0 | 0.896 | 0 | 0.894 | 0 |
| Debregeasia longifolia | 0.804 | 0 | 0.812 | 0 | 0.902 | 0 | 0.884 | 0 |
| Delonix regia | 0.752 | 0 | 0.72 | 0 | 0.787 | 0 | 0.777 | 0 |
| Desmodium elegans | 0.843 | 0 | 0.878 | 0 | 0.921 | 0 | 0.904 | 0 |
| Deutzia crenata | 0.94 | 0 | 0.96 | 0 | 0.962 | 0 | 0.956 | 0 |
| Deutzia gracilis | 0.913 | 0 | 0.859 | 0 | 0.942 | 0 | 0.928 | 0 |
| Dianella ensifolia | 0.881 | 0 | 0.865 | 0 | 0.881 | 0 | 0.871 | 0 |
| Dieffenbachia seguine | 0.827 | 0 | 0.809 | 0 | 0.919 | 0 | 0.91 | 0 |
| Dimorphotheca cuneata | 0.957 | 0 | 0.966 | 0 | 0.969 | 0 | 0.955 | 0 |
| Dimorphotheca pluvialis | 0.869 | 0 | 0.791 | 0 | 0.907 | 0 | 0.895 | 0 |
| Dimorphotheca sinuata | 0.855 | 0 | 0.882 | 0 | 0.904 | 0 | 0.891 | 0 |
| Dioscorea bulbifera | 0.776 | 0 | 0.819 | 0 | 0.867 | 0 | 0.853 | 0 |
| Dioscorea mexicana | 0.842 | 0 | 0.806 | 0 | 0.883 | 0 | 0.879 | 0 |
| Dioscorea polystachya | 0.926 | 0 | 0.749 | 0 | 0.91 | 0 | 0.918 | 0 |
| Diospyros virginiana | 0.915 | 0 | 0.928 | 0 | 0.945 | 0 | 0.931 | 0 |
| Diplocyclos palmatus | 0.833 | 0 | 0.799 | 0 | 0.865 | 0 | 0.849 | 0 |
| Dodonaea viscosa | 0.852 | 0 | 0.874 | 0 | 0.922 | 0 | 0.914 | 0 |
| Dolichandra unguis-cati | 0.729 | 0 | 0.756 | 0 | 0.857 | 0 | 0.838 | 0 |
| Dombeya burgessiae | 0.906 | 0 | 0.849 | 0 | 0.907 | 0 | 0.892 | 0 |
| Dombeya tiliacea | 0.829 | 0 | 0.795 | 0 | 0.967 | 0 | 0.95 | 0 |
| Dorotheanthus bellidiformis | 0.939 | 0 | 0.946 | 0 | 0.978 | 0 | 0.969 | 0 |
| Dorstenia contrajerva | 0.819 | 0 | 0.853 | 0 | 0.891 | 0 | 0.882 | 0 |
| Dracaena fragrans | 0.775 | 0 | 0.726 | 0 | 0.82 | 0 | 0.815 | 0 |
| Dracaena reflexa | 0.906 | 0 | 0.907 | 0 | 0.927 | 0 | 0.921 | 0 |
| Drosera aliciae | 0.906 | 0 | 0.879 | 0 | 0.928 | 0 | 0.916 | 0 |
| Drosera binata | 0.957 | 0 | 0.884 | 0 | 0.969 | 0 | 0.959 | 0 |
| Drosera capillaris | 0.908 | 0 | 0.906 | 0 | 0.914 | 0 | 0.902 | 0 |
| Echeveria secunda | 0.956 | 0 | 0.829 | 0 | 0.925 | 0 | 0.919 | 0 |
| Echinochloa polystachya | 0.778 | 0 | 0.81 | 0 | 0.88 | 0 | 0.852 | 0 |
| Echinodorus cordifolius | 0.842 | 0 | 0.862 | 0 | 0.895 | 0 | 0.886 | 0 |
| Echinodorus subalatus | 0.881 | 0 | 0.863 | 0 | 0.909 | 0 | 0.901 | 0 |
| Ehretia acuminata | 0.92 | 0 | 0.911 | 0 | 0.948 | 0 | 0.94 | 0 |
| Eichhornia azurea | 0.839 | 0 | 0.857 | 0 | 0.851 | 0 | 0.839 | 0 |
| Elaeis guineensis | 0.8 | 0 | 0.682 | 0 | 0.86 | 0 | 0.851 | 0 |
| Embothrium coccineum | 0.98 | 0 | 0.907 | 0 | 0.985 | 0 | 0.977 | 0 |
| Emilia sonchifolia | 0.771 | 0 | 0.79 | 0 | 0.867 | 0 | 0.854 | 0 |
| Empetrum rubrum | 0.572 | 33.3 | 0.859 | 0 | 0.962 | 0 | 0.956 | 0 |
| Entada phaseoloides | 0.905 | 0 | 0.855 | 0 | 0.936 | 0 | 0.948 | 36.7 |
| Epacris impressa | 0.981 | 0 | 0.981 | 0 | 0.989 | 0 | 0.975 | 0 |
| Epipremnum pinnatum | 0.758 | 0 | 0.804 | 0 | 0.821 | 0 | 0.82 | 0 |
| Eragrostis trichodes | 0.938 | 0 | 0.92 | 0 | 0.933 | 0 | 0.924 | 0 |
| Erica glandulosa | 0.992 | 0 | 0.949 | 0 | 0.964 | 0 | 0.962 | 0 |
| Eriochloa villosa | 0.888 | 0 | 0.867 | 0 | 0.923 | 0 | 0.902 | 0 |
| Erythrina crista-galli | 0.773 | 0 | 0.771 | 0 | 0.836 | 0 | 0.819 | 0 |
| Erythrina herbacea | 0.909 | 0 | 0.934 | 0 | 0.912 | 0 | 0.9 | 0 |
| Etlingera elatior | 0.832 | 0 | 0.782 | 0 | 0.84 | 0 | 0.828 | 0 |
| Eucalyptus cinerea | 0.976 | 0 | 0.902 | 0 | 0.902 | 0 | 0.9 | 0 |
| Eucalyptus cladocalyx | 0.946 | 0 | 0.928 | 0 | 0.93 | 0 | 0.924 | 0 |
| Eucalyptus cornuta | 0.935 | 0 | 0.936 | 0 | 0.965 | 0 | 0.966 | 3.3 |
| Eucalyptus dalrympleana | 0.992 | 0 | 0.996 | 0 | 0.987 | 0 | 0.981 | 0 |
| Eucalyptus goniocalyx | 0.97 | 0 | 0.966 | 0 | 0.986 | 0 | 0.977 | 0 |
| Eucalyptus leucoxylon | 0.942 | 0 | 0.953 | 0 | 0.972 | 0 | 0.97 | 0 |
| Eucalyptus nitens | 0.931 | 0 | 0.879 | 0 | 0.953 | 0 | 0.978 | 90 |
| Eucalyptus ovata | 0.962 | 0 | 0.975 | 0 | 0.984 | 0 | 0.977 | 0 |
| Eucalyptus rubida | 0.984 | 0 | 0.989 | 0 | 0.984 | 0 | 0.979 | 0 |
| Eugenia uniflora | 0.746 | 0 | 0.793 | 0 | 0.863 | 0 | 0.852 | 0 |
| Eulophia alta | 0.854 | 0 | 0.835 | 0 | 0.888 | 0 | 0.877 | 0 |
| Euonymus alatus | 0.904 | 0 | 0.9 | 0 | 0.938 | 0 | 0.926 | 0 |
| Euonymus hamiltonianus | 0.752 | 0 | 0.849 | 0 | 0.865 | 0 | 0.846 | 0 |
| Euphorbia balsamifera | 0.904 | 0 | 0.844 | 0 | 0.905 | 0 | 0.898 | 0 |
| Euphorbia leucocephala | 0.73 | 0 | 0.776 | 0 | 0.843 | 0 | 0.85 | 0 |
| Euphorbia mauritanica | 0.949 | 0 | 0.936 | 0 | 0.967 | 0 | 0.95 | 0 |
| Euphorbia tithymaloides | 0.78 | 0 | 0.798 | 0 | 0.831 | 0 | 0.817 | 0 |
| Euryops abrotanifolius | 0.96 | 0 | 0.821 | 0 | 0.973 | 0 | 0.95 | 0 |
| Euryops chrysanthemoides | 0.866 | 0 | 0.817 | 0 | 0.97 | 0 | 0.973 | 0 |
| Ficus auriculata | 0.863 | 0 | 0.715 | 0 | 0.9 | 0 | 0.885 | 0 |
| Ficus benjamina | 0.786 | 0 | 0.801 | 0 | 0.813 | 0 | 0.809 | 0 |
| Ficus drupacea | 0.911 | 0 | 0.819 | 0 | 0.872 | 0 | 0.859 | 0 |
| Ficus elastica | 0.535 | 66.7 | 0.597 | 0 | 0.717 | 6.7 | 0.714 | 6.7 |
| Ficus erecta | 0.944 | 0 | 0.939 | 0 | 0.968 | 0 | 0.96 | 0 |
| Ficus lutea | 0.835 | 0 | 0.813 | 0 | 0.863 | 0 | 0.862 | 0 |
| Ficus palmata | 0.664 | 0 | 0.834 | 0 | 0.843 | 0 | 0.823 | 0 |
| Ficus racemosa | 0.885 | 0 | 0.898 | 0 | 0.919 | 0 | 0.91 | 0 |
| Ficus virens | 0.851 | 0 | 0.86 | 0 | 0.921 | 0 | 0.885 | 0 |
| Firmiana simplex | 0.9 | 0 | 0.872 | 0 | 0.9 | 0 | 0.872 | 0 |
| Fragaria chiloensis | 0.859 | 0 | 0.878 | 0 | 0.911 | 0 | 0.903 | 0 |
| Frangula purshiana | 0.97 | 0 | 0.94 | 0 | 0.96 | 0 | 0.947 | 0 |
| Fraxinus nigra | 0.912 | 0 | 0.926 | 0 | 0.96 | 0 | 0.951 | 0 |
| Fraxinus uhdei | 0.879 | 0 | 0.863 | 0 | 0.879 | 0 | 0.866 | 0 |
| Freesia laxa | 0.89 | 0 | 0.913 | 0 | 0.938 | 0 | 0.92 | 0 |
| Fuchsia paniculata | 0.922 | 0 | 0.918 | 0 | 0.935 | 0 | 0.914 | 0 |
| Galphimia glauca | 0.888 | 0 | 0.878 | 0 | 0.891 | 0 | 0.886 | 0 |
| Galphimia gracilis | 0.771 | 0 | 0.696 | 0 | 0.843 | 0 | 0.817 | 0 |
| Garcinia livingstonei | 0.868 | 0 | 0.864 | 0 | 0.869 | 0 | 0.848 | 0 |
| Gardenia jasminoides | 0.838 | 0 | 0.86 | 0 | 0.84 | 0 | 0.828 | 0 |
| Geitonoplesium cymosum | 0.988 | 0 | 0.984 | 0 | 0.984 | 0 | 0.979 | 0 |
| Geranium incanum | 0.906 | 0 | 0.914 | 0 | 0.928 | 0 | 0.913 | 0 |
| Geranium thunbergii | 0.9 | 0 | 0.775 | 0 | 0.961 | 0 | 0.956 | 0 |
| Gerbera jamesonii | 0.805 | 0 | 0.795 | 0 | 0.87 | 3.3 | 0.851 | 3.3 |
| Gibasis pellucida | 0.838 | 0 | 0.798 | 0 | 0.865 | 0 | 0.851 | 0 |
| Gilia tricolor | 0.902 | 0 | 0.923 | 0 | 0.948 | 0 | 0.939 | 0 |
| Gladiolus papilio | 0.933 | 0 | 0.93 | 33.3 | 0.961 | 0 | 0.941 | 0 |
| Gladiolus tristis | 0.866 | 0 | 0.915 | 0 | 0.947 | 0 | 0.931 | 0 |
| Gladiolus undulatus | 0.922 | 0 | 0.925 | 0 | 0.955 | 0 | 0.942 | 0 |
| Glandularia peruviana | 0.926 | 0 | 0.918 | 0 | 0.943 | 0 | 0.929 | 0 |
| Glandularia tenera | 0.895 | 0 | 0.833 | 0 | 0.919 | 0 | 0.913 | 0 |
| Gloriosa superba | 0.781 | 0 | 0.805 | 0 | 0.876 | 0 | 0.869 | 0 |
| Gloxinia perennis | 0.855 | 0 | 0.786 | 0 | 0.905 | 0 | 0.907 | 0 |
| Gloxinia sylvatica | 0.764 | 0 | 0.811 | 0 | 0.917 | 0 | 0.903 | 0 |
| Gmelina arborea | 0.781 | 0 | 0.742 | 0 | 0.805 | 0 | 0.798 | 0 |
| Gomphrena globosa | 0.687 | 0 | 0.658 | 0 | 0.664 | 3.3 | 0.651 | 3.3 |
| Grevillea banksii | 0.706 | 33.3 | 0.861 | 0 | 0.869 | 0 | 0.859 | 0 |
| Grevillea juniperina | 0.949 | 0 | 0.935 | 0 | 0.978 | 0 | 0.972 | 0 |
| Grevillea rosmarinifolia | 0.926 | 0 | 0.919 | 0 | 0.98 | 0 | 0.969 | 0 |
| Hakea eriantha | 0.978 | 0 | 0.97 | 0 | 0.978 | 0 | 0.972 | 0 |
| Hakea laurina | 0.912 | 0 | 0.914 | 0 | 0.963 | 0 | 0.963 | 0 |
| Haloragis erecta | 0.987 | 0 | 0.939 | 0 | 0.953 | 0 | 0.945 | 0 |
| Hamelia patens | 0.781 | 0 | 0.81 | 0 | 0.892 | 0 | 0.872 | 0 |
| Hardenbergia comptoniana | 0.752 | 0 | 0.868 | 0 | 0.944 | 0 | 0.93 | 0 |
| Harpephyllum caffrum | 0.846 | 0 | 0.88 | 0 | 0.913 | 0 | 0.903 | 0 |
| Harrisia pomanensis | 0.784 | 0 | 0.923 | 0 | 0.945 | 0 | 0.894 | 0 |
| Hebenstretia dentata | 0.869 | 0 | 0.84 | 0 | 0.911 | 0 | 0.903 | 0 |
| Hedychium coronarium | 0.806 | 0 | 0.811 | 0 | 0.844 | 0 | 0.835 | 0 |
| Heimia salicifolia | 0.803 | 0 | 0.839 | 0 | 0.887 | 0 | 0.874 | 0 |
| Helanthium bolivianum | 0.801 | 0 | 0.803 | 0 | 0.86 | 0 | 0.857 | 0 |
| Helanthium tenellum | 0.691 | 0 | 0.721 | 0 | 0.815 | 0 | 0.79 | 0 |
| Helenium bigelovii | 0.939 | 0 | 0.944 | 0 | 0.956 | 0 | 0.953 | 0 |
| Helianthus angustifolius | 0.962 | 0 | 0.948 | 0 | 0.916 | 0 | 0.916 | 0 |
| Helianthus debilis | 0.735 | 0 | 0.72 | 0 | 0.817 | 0 | 0.807 | 0 |
| Helianthus giganteus | 0.878 | 0 | 0.817 | 0 | 0.886 | 0 | 0.875 | 0 |
| Helianthus salicifolius | 0.925 | 0 | 0.954 | 0 | 0.987 | 0 | 0.98 | 0 |
| Herbertia lahue | 0.915 | 0 | 0.918 | 0 | 0.927 | 0 | 0.903 | 0 |
| Hesperantha coccinea | 0.968 | 0 | 0.9 | 0 | 0.962 | 0 | 0.95 | 0 |
| Hesperantha falcata | 0.938 | 0 | 0.949 | 0 | 0.975 | 0 | 0.962 | 0 |
| Heterocentron subtriplinervium | 0.917 | 0 | 0.865 | 0 | 0.942 | 0 | 0.909 | 0 |
| Heterotis rotundifolia | 0.855 | 0 | 0.846 | 0 | 0.875 | 0 | 0.871 | 0 |
| Hevea brasiliensis | 0.846 | 0 | 0.821 | 0 | 0.882 | 0 | 0.883 | 0 |
| Hibiscus acetosella | 0.803 | 0 | 0.699 | 0 | 0.851 | 0 | 0.851 | 0 |
| Hibiscus diversifolius | 0.828 | 0 | 0.82 | 0 | 0.871 | 0 | 0.869 | 0 |
| Hibiscus mutabilis | 0.792 | 0 | 0.721 | 0 | 0.833 | 0 | 0.828 | 0 |
| Hibiscus sabdariffa | 0.735 | 0 | 0.679 | 0 | 0.797 | 0 | 0.795 | 0 |
| Hibiscus schizopetalus | 0.753 | 0 | 0.704 | 0 | 0.774 | 3.3 | 0.769 | 0 |
| Hibiscus tiliaceus | 0.858 | 0 | 0.852 | 0 | 0.898 | 0 | 0.884 | 0 |
| Hippeastrum puniceum | 0.744 | 0 | 0.642 | 0 | 0.85 | 0 | 0.833 | 0 |
| Hippobroma longiflora | 0.842 | 0 | 0.822 | 0 | 0.881 | 0 | 0.877 | 0 |
| Hiptage benghalensis | 0.909 | 0 | 0.73 | 33.3 | 0.951 | 0 | 0.909 | 0 |
| Holmskioldia sanguinea | 0.689 | 0 | 0.761 | 0 | 0.811 | 3.3 | 0.797 | 0 |
| Homalanthus populifolius | 0.937 | 0 | 0.922 | 0 | 0.95 | 0 | 0.944 | 0 |
| Homalocladium platycladum | 0.783 | 0 | 0.703 | 0 | 0.842 | 0 | 0.818 | 0 |
| Houstonia caerulea | 0.979 | 0 | 0.955 | 0 | 0.986 | 0 | 0.972 | 0 |
| Houttuynia cordata | 0.849 | 0 | 0.833 | 0 | 0.894 | 0 | 0.885 | 0 |
| Hovea pungens | 0.976 | 0 | 0.959 | 0 | 0.986 | 0 | 0.979 | 0 |
| Hovenia dulcis | 0.829 | 0 | 0.841 | 0 | 0.923 | 0 | 0.896 | 0 |
| Hoya australis | 0.841 | 0 | 0.887 | 0 | 0.92 | 0 | 0.901 | 0 |
| Hoya carnosa | 0.903 | 0 | 0.733 | 0 | 0.857 | 0 | 0.818 | 0 |
| Hura crepitans | 0.822 | 0 | 0.817 | 0 | 0.858 | 0 | 0.85 | 0 |
| Hydrangea paniculata | 0.937 | 0 | 0.915 | 0 | 0.927 | 0 | 0.919 | 0 |
| Hydrocleys nymphoides | 0.715 | 0 | 0.583 | 33.3 | 0.736 | 3.3 | 0.723 | 3.3 |
| Hydrocotyle americana | 0.93 | 0 | 0.963 | 0 | 0.955 | 0 | 0.946 | 0 |
| Hydrolea spinosa | 0.798 | 0 | 0.811 | 0 | 0.876 | 0 | 0.864 | 0 |
| Hylotelephium erythrostictum | 0.914 | 0 | 0.912 | 0 | 0.928 | 0 | 0.922 | 0 |
| Hymenocallis littoralis | 0.805 | 0 | 0.826 | 0 | 0.854 | 0 | 0.85 | 0 |
| Hypericum gramineum | 0.914 | 0 | 0.92 | 0 | 0.961 | 0 | 0.95 | 0 |
| Hypericum hypericoides | 0.912 | 0 | 0.924 | 0 | 0.95 | 0 | 0.944 | 0 |
| Hypericum patulum | 0.864 | 0 | 0.859 | 0 | 0.82 | 0 | 0.752 | 20 |
| Hypoestes aristata | 0.905 | 0 | 0.877 | 0 | 0.878 | 0 | 0.881 | 0 |
| Hypoestes phyllostachya | 0.829 | 0 | 0.864 | 0 | 0.857 | 0 | 0.837 | 0 |
| Idesia polycarpa | 0.966 | 0 | 0.836 | 0 | 0.923 | 0 | 0.923 | 0 |
| Ilex crenata | 0.938 | 0 | 0.895 | 0 | 0.928 | 0 | 0.915 | 0 |
| Ilex paraguariensis | 0.804 | 0 | 0.868 | 0 | 0.9 | 0 | 0.885 | 0 |
| Ilex rotunda | 0.931 | 0 | 0.878 | 0 | 0.948 | 0 | 0.94 | 0 |
| Indigofera tinctoria | 0.931 | 0 | 0.936 | 0 | 0.96 | 0 | 0.948 | 0 |
| Inga edulis | 0.853 | 0 | 0.849 | 0 | 0.892 | 0 | 0.886 | 0 |
| Ipomoea alba | 0.76 | 0 | 0.763 | 0 | 0.85 | 0 | 0.84 | 0 |
| Ipomoea cairica | 0.772 | 0 | 0.774 | 0 | 0.83 | 0 | 0.815 | 0 |
| Ipomoea carnea | 0.756 | 0 | 0.784 | 0 | 0.833 | 0 | 0.818 | 0 |
| Ipomoea pes caprae | 0.812 | 0 | 0.819 | 0 | 0.867 | 0 | 0.85 | 0 |
| Ipomoea tricolor | 0.67 | 0 | 0.635 | 0 | 0.795 | 0 | 0.807 | 0 |
| Iris domestica | 0.851 | 0 | 0.863 | 0 | 0.896 | 0 | 0.888 | 0 |
| Iris japonica | 0.923 | 0 | 0.921 | 0 | 0.965 | 0 | 0.954 | 0 |
| Iris sanguinea | 0.652 | 33.3 | 0.986 | 0 | 0.939 | 0 | 0.93 | 0 |
| Isotoma fluviatilis | 0.942 | 0 | 0.957 | 0 | 0.934 | 0 | 0.922 | 0 |
| Ixia polystachya | 0.939 | 0 | 0.963 | 0 | 0.973 | 0 | 0.964 | 0 |
| Ixora coccinea | 0.788 | 0 | 0.766 | 0 | 0.799 | 0 | 0.801 | 0 |
| Jasminum dichotomum | 0.877 | 0 | 0.852 | 0 | 0.876 | 0 | 0.887 | 0 |
| Jasminum fluminense | 0.808 | 0 | 0.811 | 0 | 0.864 | 0 | 0.858 | 0 |
| Jasminum grandiflorum | 0.631 | 0 | 0.57 | 33.3 | 0.72 | 3.3 | 0.697 | 3.3 |
| Jasminum multiflorum | 0.855 | 0 | 0.842 | 0 | 0.769 | 3.3 | 0.754 | 3.3 |
| Jasminum polyanthum | 0.804 | 0 | 0.843 | 0 | 0.927 | 0 | 0.918 | 0 |
| Jasminum simplicifolium | 0.94 | 0 | 0.852 | 0 | 0.939 | 0 | 0.931 | 0 |
| Justicia betonica | 0.838 | 0 | 0.821 | 0 | 0.88 | 0 | 0.874 | 0 |
| Justicia brandegeeana | 0.683 | 0 | 0.907 | 0 | 0.8 | 0 | 0.772 | 0 |
| Justicia spicigera | 0.754 | 0 | 0.736 | 0 | 0.857 | 0 | 0.85 | 0 |
| Kalanchoe crenata | 0.884 | 0 | 0.749 | 33.3 | 0.841 | 0 | 0.841 | 0 |
| Kalopanax septemlobus | 0.9 | 0 | 0.87 | 0 | 0.941 | 0 | 0.936 | 0 |
| Kennedia rubicunda | 0.987 | 0 | 0.977 | 0 | 0.979 | 0 | 0.972 | 0 |
| Kigelia africana | 0.762 | 0 | 0.778 | 0 | 0.83 | 0 | 0.817 | 0 |
| Kolkwitzia amabilis | 0.913 | 0 | 0.881 | 0 | 0.921 | 0 | 0.919 | 0 |
| Kummerowia stipulacea | 0.917 | 0 | 0.901 | 0 | 0.964 | 0 | 0.96 | 0 |
| Kummerowia striata | 0.932 | 0 | 0.897 | 0 | 0.954 | 0 | 0.947 | 0 |
| Kunzea ambigua | 0.949 | 0 | 0.929 | 0 | 0.973 | 0 | 0.97 | 0 |
| Kunzea ericoides | 0.98 | 0 | 0.978 | 0 | 0.983 | 0 | 0.981 | 0 |
| Lablab purpureus | 0.752 | 0 | 0.762 | 0 | 0.804 | 0 | 0.799 | 0 |
| Laelia rubescens | 0.863 | 0 | 0.927 | 0 | 0.881 | 0 | 0.856 | 0 |
| Lagerstroemia speciosa | 0.836 | 0 | 0.763 | 0 | 0.873 | 0 | 0.853 | 0 |
| Lampranthus spectabilis | 0.787 | 0 | 0.751 | 0 | 0.91 | 0 | 0.887 | 0 |
| Lawsonia inermis | 0.762 | 0 | 0.755 | 0 | 0.817 | 0 | 0.817 | 0 |
| Leonotis leonurus | NaN | 100 | 0.944 | 0 | 0.931 | 0 | 0.915 | 13.3 |
| Leonotis ocymifolia | 0.921 | 0 | 0.916 | 0 | 0.916 | 0 | 0.909 | 0 |
| Leonurus japonicus | 0.731 | 0 | 0.7 | 0 | 0.823 | 0 | 0.802 | 0 |
| Leonurus sibiricus | 0.682 | 0 | 0.705 | 0 | 0.829 | 0 | 0.809 | 0 |
| Leptospermum polygalifolium | 0.978 | 0 | 0.989 | 0 | 0.987 | 0 | 0.982 | 0 |
| Lespedeza bicolor | 0.867 | 0 | 0.814 | 0 | 0.928 | 0 | 0.911 | 0 |
| Lespedeza cyrtobotrya | 0.982 | 0 | 0.969 | 0 | 0.986 | 0 | 0.982 | 0 |
| Lespedeza thunbergii | 0.881 | 0 | 0.897 | 0 | 0.939 | 0 | 0.918 | 0 |
| Leucophyllum frutescens | 0.755 | 0 | 0.94 | 0 | 0.956 | 0 | 0.935 | 0 |
| Ligustrum obtusifolium | 0.883 | 0 | 0.889 | 0 | 0.944 | 0 | 0.935 | 0 |
| Ligustrum tschonoskii | 0.959 | 0 | 0.923 | 0 | 0.968 | 0 | 0.963 | 0 |
| Lilium formosanum | 0.95 | 0 | 0.937 | 0 | 0.946 | 0 | 0.942 | 0 |
| Limnobium laevigatum | 0.691 | 0 | 0.795 | 0 | 0.8 | 0 | 0.788 | 0 |
| Limnocharis flava | 0.753 | 0 | 0.807 | 0 | 0.84 | 0 | 0.833 | 0 |
| Linaria maroccana | 0.814 | 0 | 0.832 | 0 | 0.865 | 0 | 0.837 | 0 |
| Linum grandiflorum | 0.891 | 0 | 0.894 | 0 | 0.923 | 0 | 0.911 | 0 |
| Liriope muscari | 0.884 | 0 | 0.815 | 0 | 0.911 | 0 | 0.898 | 0 |
| Liriope spicata | 0.877 | 0 | 0.863 | 0 | 0.915 | 0 | 0.917 | 0 |
| Livistona australis | 0.938 | 0 | 0.932 | 0 | 0.961 | 0 | 0.958 | 0 |
| Lobelia cardinalis | 0.727 | 0 | 0.786 | 0 | 0.876 | 0 | 0.812 | 0 |
| Lobelia inflata | 0.928 | 0 | 0.93 | 0 | 0.959 | 0 | 0.952 | 0 |
| Lonicera sempervirens | 0.96 | 0 | 0.937 | 0 | 0.957 | 0 | 0.935 | 0 |
| Lophostemon confertus | 0.953 | 0 | 0.936 | 0 | 0.963 | 0 | 0.957 | 0 |
| Ludwigia alternifolia | 0.962 | 0 | 0.958 | 0 | 0.979 | 0 | 0.977 | 0 |
| Ludwigia octovalvis | 0.724 | 0 | 0.76 | 0 | 0.865 | 0 | 0.836 | 0 |
| Ludwigia peruviana | 0.776 | 0 | 0.79 | 0 | 0.848 | 0 | 0.837 | 0 |
| Luffa cylindrica | 0.697 | 0 | 0.669 | 0 | 0.794 | 0 | 0.794 | 0 |
| Luma apiculata | 0.841 | 0 | 0.842 | 0 | 0.91 | 0 | 0.902 | 0 |
| Lupinus mexicanus | 0.95 | 0 | 0.891 | 0 | 0.947 | 0 | 0.94 | 0 |
| Lycianthes rantonnetii | 0.638 | 0 | 0.66 | 33.3 | 0.73 | 0 | 0.708 | 3.3 |
| Lycoris radiata | 0.917 | 0 | 0.891 | 0 | 0.993 | 0 | 0.963 | 0 |
| Lysimachia japonica | 0.945 | 0 | 0.974 | 0 | 0.958 | 0 | 0.946 | 0 |
| Maackia amurensis | 0.968 | 0 | 0.926 | 0 | 0.923 | 0 | 0.918 | 0 |
| Magnolia grandiflora | 0.744 | 0 | 0.722 | 0 | 0.789 | 0 | 0.777 | 0 |
| Magnolia kobus | 0.586 | 33.3 | 0.681 | 0 | 0.881 | 0 | 0.869 | 0 |
| Magnolia obovata | 0.866 | 0 | 0.833 | 0 | 0.93 | 0 | 0.924 | 0 |
| Malephora crocea | 0.924 | 0 | 0.946 | 0 | 0.964 | 0 | 0.958 | 0 |
| Mallotus philippensis | 0.783 | 0 | 0.799 | 0 | 0.868 | 0 | 0.847 | 0 |
| Malpighia emarginata | 0.8 | 0 | 0.776 | 0 | 0.846 | 0 | 0.833 | 0 |
| Malpighia glabra | 0.779 | 0 | 0.805 | 0 | 0.867 | 0 | 0.851 | 0 |
| Malpighia mexicana | 0.874 | 0 | 0.841 | 0 | 0.945 | 0 | 0.933 | 0 |
| Malus prunifolia | 0.876 | 0 | 0.879 | 0 | 0.9 | 0 | 0.889 | 0 |
| Malva assurgentiflora | 0.954 | 0 | 0.917 | 0 | 0.957 | 0 | 0.942 | 0 |
| Malvaviscus arboreus | 0.788 | 0 | 0.809 | 0 | 0.886 | 0 | 0.85 | 0 |
| Mammea americana | 0.852 | 0 | 0.825 | 0 | 0.813 | 0 | 0.802 | 0 |
| Mandevilla laxa | 0.823 | 0 | 0.832 | 0 | 0.933 | 0 | 0.924 | 0 |
| Mangifera indica | 0.743 | 0 | 0.769 | 0 | 0.825 | 0 | 0.807 | 0 |
| Manilkara zapota | 0.815 | 0 | 0.839 | 0 | 0.867 | 0 | 0.863 | 0 |
| Maranta arundinacea | 0.838 | 0 | 0.856 | 0 | 0.868 | 0 | 0.868 | 0 |
| Margyricarpus pinnatus | 0.877 | 0 | 0.81 | 0 | 0.917 | 0 | 0.909 | 0 |
| Martynia annua | 0.789 | 0 | 0.804 | 0 | 0.844 | 0 | 0.841 | 0 |
| Maurandya antirrhiniflora | 0.827 | 0 | 0.849 | 0 | 0.899 | 0 | 0.889 | 0 |
| Mazus pumilus | 0.847 | 0 | 0.863 | 0 | 0.903 | 0 | 0.902 | 0 |
| Melaleuca hypericifolia | 0.976 | 0 | 0.906 | 0 | 0.978 | 0 | 0.969 | 0 |
| Melastoma malabathricum | 0.799 | 0 | 0.858 | 0 | 0.901 | 0 | 0.874 | 0 |
| Melianthus major | 0.915 | 0 | 0.81 | 0 | 0.93 | 0 | 0.927 | 0 |
| Melinis repens | 0.679 | 0 | 0.708 | 0 | 0.86 | 0 | 0.812 | 0 |
| Melothria pendula | 0.729 | 0 | 0.773 | 0 | 0.86 | 0 | 0.831 | 0 |
| Miconia calvescens | 0.849 | 0 | 0.856 | 0 | 0.888 | 0 | 0.883 | 0 |
| Micranthemum umbrosum | 0.731 | 0 | 0.764 | 0 | 0.846 | 0 | 0.82 | 0 |
| Mimosa pigra | 0.762 | 0 | 0.773 | 0 | 0.875 | 0 | 0.861 | 0 |
| Mimusops elengi | 0.888 | 0 | 0.867 | 0 | 0.904 | 0 | 0.891 | 60 |
| Molineria capitulata | 0.774 | 0 | 0.66 | 0 | 0.797 | 0 | 0.792 | 0 |
| Momordica balsamina | 0.75 | 0 | 0.775 | 0 | 0.838 | 0 | 0.821 | 0 |
| Momordica charantia | 0.742 | 0 | 0.751 | 0 | 0.841 | 0 | 0.829 | 0 |
| Momordica cochinchinensis | 0.908 | 0 | 0.801 | 0 | 0.907 | 0 | 0.883 | 0 |
| Monarda fistulosa | 0.824 | 0 | 0.863 | 0 | 0.91 | 0 | 0.879 | 0 |
| Monarda punctata | 0.918 | 0 | 0.926 | 0 | 0.947 | 0 | 0.946 | 0 |
| Monochoria vaginalis | 0.868 | 0 | 0.843 | 0 | 0.9 | 0 | 0.884 | 0 |
| Moraea flaccida | NaN | 100 | 0.947 | 0 | 0.969 | 0 | 0.953 | 0 |
| Moraea fugax | 0.942 | 0 | 0.925 | 0 | 0.975 | 0 | 0.949 | 0 |
| Moraea miniata | NaN | 100 | 0.922 | 0 | 0.951 | 0 | 0.948 | 20 |
| Moraea polystachya | 0.968 | 0 | 0.947 | 0 | 0.94 | 0 | 0.923 | 0 |
| Moraea setifolia | 0.954 | 0 | 0.948 | 0 | 0.969 | 0 | 0.963 | 0 |
| Morinda citrifolia | 0.877 | 0 | 0.885 | 0 | 0.895 | 0 | 0.89 | 0 |
| Moringa oleifera | 0.769 | 0 | 0.746 | 0 | 0.806 | 0 | 0.813 | 0 |
| Mucuna pruriens | 0.791 | 0 | 0.813 | 0 | 0.848 | 0 | 0.848 | 0 |
| Muehlenbeckia axillaris | 0.994 | 0 | 0.983 | 0 | 0.984 | 0 | 0.978 | 0 |
| Mukia maderaspatana | 0.667 | 0 | 0.748 | 0 | 0.857 | 0 | 0.838 | 0 |
| Musa acuminata | 0.57 | 0 | 0.831 | 0 | 0.789 | 0 | 0.789 | 3.3 |
| Myrica rubra | 0.961 | 0 | 0.943 | 0 | 0.964 | 0 | 0.96 | 0 |
| Myrmecophila tibicinis | 0.949 | 0 | 0.936 | 0 | 0.939 | 0 | 0.919 | 0 |
| Nandina domestica | 0.855 | 0 | 0.832 | 0 | 0.884 | 0 | 0.864 | 0 |
| Nepeta racemosa | 0.851 | 0 | 0.902 | 0 | 0.925 | 0 | 0.918 | 0 |
| Neptunia oleracea | 0.833 | 0 | 0.847 | 0 | 0.845 | 0 | 0.833 | 0 |
| Neptunia plena | 0.831 | 0 | 0.822 | 0 | 0.838 | 0 | 0.829 | 0 |
| Nertera granadensis | 0.927 | 0 | 0.911 | 0 | 0.949 | 0 | 0.944 | 0 |
| Nicotiana acuminata | 0.915 | 0 | 0.906 | 0 | 0.928 | 0 | 0.915 | 0 |
| Nicotiana longiflora | 0.644 | 0 | 0.677 | 0 | 0.819 | 0 | 0.796 | 0 |
| Nicotiana sylvestris | 0.718 | 0 | 0.743 | 0 | 0.847 | 0 | 0.833 | 0 |
| Nothoscordum bivalve | 0.846 | 0 | 0.89 | 0 | 0.891 | 0 | 0.869 | 0 |
| Nymphaea nouchali | 0.86 | 0 | 0.852 | 0 | 0.889 | 0 | 0.875 | 0 |
| Nymphaea odorata | 0.778 | 0 | 0.822 | 0 | 0.879 | 0 | 0.839 | 0 |
| Ochroma pyramidale | 0.844 | 0 | 0.836 | 0 | 0.88 | 0 | 0.872 | 0 |
| Ocimum americanum | 0.734 | 0 | 0.758 | 0 | 0.812 | 0 | 0.793 | 0 |
| Ocimum gratissimum | 0.775 | 0 | 0.762 | 0 | 0.845 | 0 | 0.837 | 0 |
| Ocimum kilimandscharicum | 0.891 | 0 | 0.844 | 0 | 0.887 | 0 | 0.871 | 0 |
| Ocimum tenuiflorum | 0.821 | 0 | 0.815 | 0 | 0.865 | 0 | 0.854 | 0 |
| Odontonema tubaeforme | 0.817 | 0 | 0.813 | 0 | 0.86 | 0 | 0.861 | 0 |
| Oeceoclades maculata | 0.804 | 0 | 0.767 | 0 | 0.845 | 0 | 0.837 | 0 |
| Oenothera drummondii | 0.63 | 0 | 0.865 | 0 | 0.899 | 0 | 0.897 | 0 |
| Oenothera perennis | 0.951 | 0 | 0.915 | 0 | 0.946 | 0 | 0.928 | 0 |
| Opuntia aurantiaca | 0.959 | 0 | 0.944 | 0 | 0.978 | 0 | 0.969 | 0 |
| Opuntia basilaris | 0.777 | 0 | 0.912 | 0 | 0.948 | 0 | 0.926 | 0 |
| Opuntia humifusa | 0.92 | 0 | 0.941 | 0 | 0.916 | 0 | 0.911 | 0 |
| Opuntia leucotricha | 0.91 | 0 | 0.805 | 0 | 0.948 | 0 | 0.939 | 0 |
| Opuntia polyacantha | 0.911 | 0 | 0.923 | 0 | 0.927 | 0 | 0.917 | 0 |
| Ornithogalum thyrsoides | 0.877 | 0 | 0.84 | 0 | 0.933 | 0 | 0.909 | 0 |
| Orthrosanthus chimboracensis | 0.917 | 0 | 0.888 | 0 | 0.96 | 0 | 0.948 | 0 |
| Osmanthus heterophyllus | 0.898 | 0 | 0.907 | 0 | 0.937 | 0 | 0.92 | 0 |
| Osteospermum ecklonis | 0.896 | 0 | 0.881 | 0 | 0.922 | 0 | 0.911 | 0 |
| Oxalis depressa | 0.939 | 0 | 0.933 | 0 | 0.949 | 0 | 0.932 | 0 |
| Oxalis spiralis | 0.932 | 0 | 0.91 | 0 | 0.943 | 0 | 0.943 | 0 |
| Oxalis tuberosa | 0.915 | 0 | 0.857 | 0 | 0.93 | 0 | 0.933 | 0 |
| Pachira aquatica | 0.845 | 0 | 0.854 | 0 | 0.893 | 0 | 0.883 | 0 |
| Paeonia lactiflora | 0.854 | 0 | 0.855 | 33.3 | 0.908 | 0 | 0.886 | 0 |
| Pandanus tectorius | 0.918 | 0 | 0.933 | 0 | 0.889 | 0 | 0.888 | 0 |
| Pandorea jasminoides | 0.843 | 0 | 0.829 | 0 | 0.955 | 0 | 0.949 | 0 |
| Pandorea pandorana | 0.952 | 0 | 0.958 | 0 | 0.975 | 0 | 0.964 | 0 |
| Papaver aculeatum | 0.933 | 0 | 0.922 | 0 | 0.957 | 0 | 0.954 | 0 |
| Papaver nudicaule | 0.779 | 0 | 0.839 | 0 | 0.91 | 0 | 0.896 | 0 |
| Papaver orientale | 0.894 | 0 | 0.908 | 0 | 0.942 | 0 | 0.926 | 0 |
| Parmentiera aculeata | 0.852 | 0 | 0.859 | 0 | 0.893 | 0 | 0.888 | 0 |
| Parochetus communis | 0.888 | 0 | 0.897 | 0 | 0.906 | 0 | 0.892 | 0 |
| Passiflora amethystina | 0.869 | 0 | 0.858 | 0 | 0.938 | 0 | 0.931 | 0 |
| Passiflora coccinea | 0.853 | 0 | 0.852 | 0 | 0.904 | 0 | 0.905 | 0 |
| Passiflora foetida | 0.734 | 0 | 0.756 | 0 | 0.861 | 0 | 0.832 | 0 |
| Passiflora laurifolia | 0.878 | 0 | 0.833 | 0 | 0.931 | 0 | 0.933 | 0 |
| Passiflora ligularis | 0.836 | 0 | 0.814 | 0 | 0.91 | 0 | 0.904 | 0 |
| Passiflora mixta | 0.96 | 0 | 0.949 | 0 | 0.978 | 0 | 0.975 | 0 |
| Passiflora quadrangularis | 0.861 | 0 | 0.848 | 0 | 0.862 | 0 | 0.844 | 0 |
| Passiflora vitifolia | 0.889 | 0 | 0.865 | 0 | 0.919 | 0 | 0.914 | 0 |
| Pavonia hastata | 0.91 | 0 | 0.894 | 0 | 0.963 | 0 | 0.948 | 0 |
| Pelargonium alchemilloides | 0.967 | 0 | 0.965 | 0 | 0.941 | 0 | 0.936 | 0 |
| Pelargonium grossularioides | 0.882 | 0 | 0.88 | 0 | 0.931 | 0 | 0.927 | 0 |
| Pennisetum alopecuroides | 0.858 | 0 | 0.878 | 0 | 0.922 | 0 | 0.912 | 0 |
| Pennisetum orientale | 0.774 | 0 | 0.776 | 0 | 0.817 | 0 | 0.815 | 0 |
| Penstemon gentianoides | 0.961 | 0 | 0.872 | 0 | 0.936 | 0 | 0.93 | 0 |
| Pentas lanceolata | 0.81 | 0 | 0.834 | 0 | 0.832 | 0 | 0.829 | 0 |
| Peperomia obtusifolia | 0.816 | 0 | 0.84 | 0 | 0.893 | 0 | 0.892 | 0 |
| Pereskia aculeata | 0.885 | 0 | 0.875 | 0 | 0.902 | 0 | 0.884 | 0 |
| Perovskia atriplicifolia | 0.551 | 33.3 | 0.636 | 0 | 0.794 | 10 | 0.788 | 6.7 |
| Persea americana | 0.711 | 0 | 0.734 | 0 | 0.833 | 0 | 0.799 | 0 |
| Petrea volubilis | 0.803 | 0 | 0.825 | 0 | 0.871 | 0 | 0.866 | 26.7 |
| Petunia axillaris | 0.802 | 0 | 0.788 | 0 | 0.866 | 0 | 0.848 | 0 |
| Petunia integrifolia | 0.928 | 0 | 0.945 | 0 | 0.89 | 0 | 0.875 | 0 |
| Philadelphus mexicanus | 0.903 | 0 | 0.826 | 0 | 0.945 | 0 | 0.939 | 0 |
| Philadelphus pubescens | 0.86 | 0 | 0.9 | 0 | 0.925 | 0 | 0.92 | 0 |
| Philodendron bipinnatifidum | 0.836 | 0 | 0.785 | 0 | 0.856 | 0 | 0.833 | 0 |
| Philodendron ornatum | 0.894 | 0 | 0.885 | 0 | 0.93 | 0 | 0.926 | 0 |
| Phlox drummondii | 0.823 | 0 | 0.814 | 0 | 0.836 | 0 | 0.833 | 0 |
| Phoenix reclinata | 0.777 | 0 | 0.771 | 0 | 0.855 | 0 | 0.854 | 0 |
| Photinia glabra | 0.882 | 0 | 0.873 | 0 | 0.889 | 0 | 0.883 | 0 |
| Phyllanthus amarus | 0.794 | 0 | 0.798 | 0 | 0.857 | 0 | 0.847 | 63.3 |
| Phyllanthus emblica | 0.787 | 0 | 0.83 | 0 | 0.886 | 0 | 0.872 | 0 |
| Pimenta dioica | 0.83 | 0 | 0.813 | 0 | 0.872 | 0 | 0.863 | 0 |
| Pinellia ternata | 0.92 | 0 | 0.804 | 0 | 0.933 | 0 | 0.913 | 0 |
| Piper aduncum | 0.825 | 0 | 0.816 | 0 | 0.896 | 0 | 0.889 | 0 |
| Piscidia piscipula | 0.876 | 0 | 0.872 | 0 | 0.892 | 0 | 0.896 | 0 |
| Pistacia chinensis | 0.77 | 0 | 0.775 | 0 | 0.852 | 0 | 0.827 | 0 |
| Pithecellobium dulce | 0.732 | 0 | 0.766 | 0 | 0.818 | 0 | 0.802 | 0 |
| Pittosporum bicolor | 0.963 | 0 | 0.963 | 0 | 0.982 | 0 | 0.972 | 0 |
| Pittosporum eugenioides | 0.965 | 0 | 0.928 | 0 | 0.988 | 0 | 0.983 | 0 |
| Plantago rugelii | 0.956 | 0 | 0.977 | 0 | 0.973 | 0 | 0.97 | 33.3 |
| Plectranthus amboinicus | 0.777 | 0 | 0.799 | 0 | 0.854 | 0 | 0.839 | 0 |
| Plectranthus ciliatus | 0.989 | 0 | 0.954 | 0 | 0.976 | 0 | 0.959 | 0 |
| Plectranthus scutellarioides | 0.836 | 0 | 0.863 | 0 | 0.892 | 0 | 0.877 | 0 |
| Plectranthus verticillatus | 0.882 | 0 | 0.876 | 0 | 0.897 | 0 | 0.876 | 0 |
| Plumbago zeylanica | 0.695 | 0 | 0.73 | 0 | 0.846 | 0 | 0.816 | 0 |
| Plumeria obtusa | 0.93 | 0 | 0.952 | 0 | 0.932 | 0 | 0.917 | 0 |
| Plumeria rubra | 0.727 | 0 | 0.76 | 0 | 0.814 | 0 | 0.795 | 0 |
| Polygala lancifolia | 0.864 | 0 | 0.894 | 0 | 0.983 | 0 | 0.952 | 0 |
| Polygala senega | 0.711 | 33.3 | 0.938 | 0 | 0.9 | 0 | 0.891 | 0 |
| Pomaderris lanigera | 0.971 | 0 | 0.963 | 0 | 0.981 | 0 | 0.978 | 0 |
| Populus acuminata | 0.703 | 33.3 | 0.831 | 0 | 0.987 | 0 | 0.969 | 0 |
| Portulacaria afra | 0.955 | 0 | 0.987 | 0 | 0.93 | 0 | 0.903 | 0 |
| Pouteria caimito | 0.877 | 0 | 0.875 | 0 | 0.922 | 0 | 0.93 | 0 |
| Pratia repens | 0.999 | 0 | 0.944 | 0 | 0.975 | 0 | 0.958 | 0 |
| Prosopis chilensis | 0.649 | 0 | 0.749 | 0 | 0.864 | 0 | 0.846 | 0 |
| Prosopis juliflora | 0.624 | 0 | 0.651 | 0 | 0.766 | 0 | 0.761 | 0 |
| Prunus munsoniana | 0.895 | 0 | 0.874 | 0 | 0.97 | 0 | 0.97 | 0 |
| Prunus pumila | 0.902 | 0 | 0.928 | 0 | 0.928 | 0 | 0.921 | 0 |
| Prunus salicina | 0.881 | 0 | 0.798 | 0 | 0.887 | 0 | 0.881 | 0 |
| Psoralea pinnata | 0.89 | 0 | 0.87 | 0 | 0.935 | 0 | 0.933 | 0 |
| Pueraria montana | 0.814 | 0 | 0.811 | 0 | 0.862 | 0 | 0.851 | 0 |
| Pyracantha fortuneana | 0.897 | 0 | 0.935 | 0 | 0.884 | 0 | 0.87 | 0 |
| Pyracantha koidzumii | 0.644 | 33.3 | 0.645 | 0 | 0.841 | 0 | 0.807 | 0 |
| Pyrus calleryana | 0.818 | 0 | 0.771 | 0 | 0.85 | 0 | 0.835 | 0 |
| Pyrus pyrifolia | 0.885 | 0 | 0.846 | 0 | 0.892 | 0 | 0.873 | 0 |
| Quassia amara | 0.834 | 0 | 0.836 | 0 | 0.88 | 0 | 0.876 | 0 |
| Quercus acutissima | 0.875 | 0 | 0.873 | 0 | 0.917 | 0 | 0.909 | 0 |
| Rauvolfia tetraphylla | 0.83 | 0 | 0.853 | 0 | 0.87 | 0 | 0.859 | 0 |
| Rauvolfia vomitoria | 0.864 | 0 | 0.883 | 0 | 0.929 | 0 | 0.917 | 0 |
| Rhamnus japonica | 0.939 | 0 | 0.921 | 0 | 0.976 | 0 | 0.976 | 0 |
| Rhaphiolepis indica | 0.898 | 0 | 0.823 | 0 | 0.915 | 0 | 0.91 | 0 |
| Rheum rhabarbarum | 0.904 | 0 | 0.916 | 0 | 0.95 | 0 | 0.943 | 0 |
| Rhipsalis baccifera | 0.828 | 0 | 0.832 | 0 | 0.905 | 0 | 0.894 | 0 |
| Rhodanthe chlorocephala | 0.892 | 0 | 0.916 | 0 | 0.932 | 0 | 0.922 | 0 |
| Rhododendron japonicum | 0.737 | 0 | 0.769 | 0 | 0.942 | 0 | 0.925 | 0 |
| Romneya coulteri | 0.907 | 0 | 0.873 | 0 | 0.913 | 0 | 0.903 | 0 |
| Romulea flava | 0.959 | 0 | 0.958 | 0 | 0.949 | 0 | 0.936 | 0 |
| Rosa banksiae | 0.902 | 0 | 0.847 | 0 | 0.92 | 0 | 0.89 | 0 |
| Rosa chinensis | 0.832 | 0 | 0.758 | 0 | 0.819 | 0 | 0.767 | 0 |
| Rosenbergiodendron formosum | 0.855 | 0 | 0.815 | 0 | 0.918 | 0 | 0.918 | 0 |
| Rubus ellipticus | 0.909 | 0 | 0.88 | 0 | 0.905 | 0 | 0.889 | 0 |
| Rubus rosifolius | 0.875 | 0 | 0.903 | 0 | 0.934 | 0 | 0.931 | 0 |
| Rudbeckia triloba | 0.944 | 0 | 0.941 | 33.3 | 0.976 | 0 | 0.971 | 0 |
| Ruellia brevifolia | 0.802 | 0 | 0.828 | 0 | 0.891 | 0 | 0.868 | 0 |
| Ruellia tuberosa | 0.827 | 0 | 0.845 | 0 | 0.815 | 0 | 0.809 | 0 |
| Russelia equisetiformis | 0.661 | 0 | 0.751 | 0 | 0.853 | 0 | 0.817 | 0 |
| Russelia sarmentosa | 0.853 | 0 | 0.874 | 0 | 0.891 | 0 | 0.882 | 0 |
| Sageretia thea | 0.702 | 0 | 0.818 | 0 | 0.841 | 0 | 0.835 | 0 |
| Sagittaria graminea | 0.856 | 0 | 0.888 | 0 | 0.929 | 0 | 0.921 | 0 |
| Sagittaria montevidensis | 0.8 | 0 | 0.791 | 0 | 0.875 | 0 | 0.856 | 0 |
| Salix gracilistyla | 0.957 | 0 | 0.945 | 0 | 0.991 | 0 | 0.988 | 0 |
| Salix humboldtiana | 0.741 | 0 | 0.777 | 0 | 0.855 | 0 | 0.835 | 0 |
| Salix nigra | 0.901 | 0 | 0.896 | 0 | 0.915 | 0 | 0.901 | 0 |
| Salvia africana lutea | 0.946 | 0 | 0.935 | 0 | 0.997 | 0 | 0.985 | 0 |
| Salvia leucantha | 0.651 | 33.3 | 0.688 | 0 | 0.813 | 0 | 0.802 | 0 |
| Salvia leucophylla | 0.948 | 0 | 0.901 | 0 | 0.925 | 0 | 0.908 | 0 |
| Salvia microphylla | 0.875 | 0 | 0.881 | 0 | 0.908 | 0 | 0.902 | 0 |
| Salvia plebeia | 0.829 | 0 | 0.8 | 0 | 0.851 | 0 | 0.837 | 0 |
| Salvia splendens | 0.516 | 66.7 | 0.569 | 33.3 | 0.782 | 0 | 0.751 | 0 |
| Sansevieria hyacinthoides | 0.818 | 0 | 0.869 | 0 | 0.867 | 0 | 0.857 | 0 |
| Santalum album | 0.862 | 0 | 0.839 | 0 | 0.888 | 0 | 0.891 | 0 |
| Sanvitalia procumbens | 0.772 | 0 | 0.889 | 0 | 0.909 | 0 | 0.888 | 0 |
| Sauropus androgynus | 0.832 | 0 | 0.834 | 0 | 0.877 | 0 | 0.871 | 0 |
| Scadoxus multiflorus | 0.778 | 0 | 0.803 | 0 | 0.851 | 0 | 0.84 | 0 |
| Scaevola taccada | 0.885 | 0 | 0.896 | 0 | 0.906 | 0 | 0.903 | 0 |
| Schefflera arboricola | 0.89 | 0 | 0.83 | 0 | 0.933 | 0 | 0.918 | 0 |
| Schisandra chinensis | 0.71 | 0 | 0.77 | 0 | 0.951 | 0 | 0.945 | 0 |
| Schotia brachypetala | 0.951 | 0 | 0.949 | 0 | 0.975 | 0 | 0.955 | 0 |
| Senecio radicans | 0.943 | 0 | 0.961 | 0 | 0.961 | 0 | 0.942 | 0 |
| Senecio tamoides | 0.871 | 0 | 0.944 | 0 | 0.969 | 0 | 0.956 | 0 |
| Senna artemisioides | 0.927 | 0 | 0.943 | 0 | 0.976 | 0 | 0.967 | 0 |
| Senna italica | 0.806 | 0 | 0.846 | 0 | 0.899 | 0 | 0.88 | 0 |
| Senna siamea | 0.792 | 0 | 0.785 | 0 | 0.831 | 0 | 0.82 | 0 |
| Sesbania grandiflora | 0.853 | 0 | 0.712 | 0 | 0.842 | 0 | 0.825 | 0 |
| Sesbania sesban | 0.762 | 0 | 0.759 | 0 | 0.867 | 0 | 0.855 | 0 |
| Sisyrinchium atlanticum | 0.893 | 0 | 0.946 | 0 | 0.946 | 0 | 0.934 | 0 |
| Sisyrinchium micranthum | 0.867 | 0 | 0.893 | 0 | 0.91 | 0 | 0.906 | 0 |
| Sisyrinchium mucronatum | 0.916 | 0 | 0.869 | 0 | 0.948 | 0 | 0.924 | 0 |
| Solandra maxima | 0.85 | 0 | 0.763 | 33.3 | 0.818 | 0 | 0.791 | 0 |
| Solanum aviculare | 0.91 | 0 | 0.933 | 0 | 0.949 | 0 | 0.936 | 0 |
| Solanum betaceum | 0.873 | 0 | 0.782 | 0 | 0.826 | 0 | 0.836 | 0 |
| Solanum capsicoides | 0.802 | 0 | 0.794 | 0 | 0.855 | 0 | 0.85 | 0 |
| Solanum chrysotrichum | 0.873 | 0 | 0.625 | 33.3 | 0.92 | 0 | 0.9 | 0 |
| Solanum lanceifolium | 0.832 | 0 | 0.827 | 0 | 0.87 | 0 | 0.869 | 0 |
| Solanum mammosum | 0.828 | 0 | 0.814 | 0 | 0.845 | 0 | 0.834 | 0 |
| Solanum quitoense | 0.925 | 0 | 0.885 | 0 | 0.9 | 0 | 0.91 | 0 |
| Solanum retroflexum | 0.925 | 0 | 0.901 | 0 | 0.935 | 0 | 0.924 | 0 |
| Solanum seaforthianum | 0.757 | 0 | 0.789 | 0 | 0.846 | 0 | 0.834 | 0 |
| Solanum sessiliflorum | 0.87 | 0 | 0.858 | 0 | 0.925 | 0 | 0.918 | 0 |
| Solanum wendlandii | 0.813 | 0 | 0.878 | 0 | 0.898 | 0 | 0.857 | 0 |
| Solidago altissima | 0.801 | 0 | 0.824 | 0 | 0.885 | 0 | 0.864 | 0 |
| Solidago ptarmicoides | 0.731 | 33.3 | 0.847 | 0 | 0.857 | 0 | 0.857 | 0 |
| Sophora tetraptera | 0.888 | 0 | 0.887 | 0 | 0.956 | 0 | 0.947 | 0 |
| Sorbus alnifolia | 0.932 | 0 | 0.843 | 0 | 0.929 | 0 | 0.926 | 0 |
| Sparaxis grandiflora | 0.94 | 0 | 0.965 | 0 | 0.987 | 0 | 0.973 | 0 |
| Spathiphyllum cannifolium | 0.903 | 0 | 0.904 | 0 | 0.929 | 0 | 0.932 | 0 |
| Spathodea campanulata | 0.803 | 0 | 0.781 | 0 | 0.836 | 0 | 0.836 | 0 |
| Spathoglottis plicata | 0.898 | 0 | 0.709 | 0 | 0.91 | 0 | 0.91 | 0 |
| Spiraea prunifolia | 0.802 | 0 | 0.84 | 0 | 0.886 | 0 | 0.874 | 0 |
| Spondias dulcis | 0.795 | 0 | 0.724 | 0 | 0.881 | 0 | 0.857 | 0 |
| Spondias mombin | 0.829 | 0 | 0.824 | 0 | 0.887 | 0 | 0.877 | 0 |
| Stachytarpheta jamaicensis | 0.837 | 0 | 0.851 | 0 | 0.884 | 0 | 0.881 | 6.7 |
| Stachytarpheta mutabilis | 0.806 | 0 | 0.679 | 33.3 | 0.819 | 0 | 0.809 | 0 |
| Stapelia grandiflora | 0.953 | 0 | 0.946 | 0 | 0.948 | 0 | 0.929 | 3.3 |
| Stenocarpus sinuatus | 0.685 | 0 | 0.857 | 0 | 0.895 | 0 | 0.869 | 0 |
| Sterculia apetala | 0.819 | 0 | 0.837 | 0 | 0.897 | 0 | 0.887 | 0 |
| Stigmaphyllon ellipticum | 0.891 | 0 | 0.877 | 0 | 0.875 | 0 | 0.874 | 0 |
| Stipa tenuissima | 0.875 | 0 | 0.85 | 0 | 0.886 | 0 | 0.881 | 0 |
| Streptosolen jamesonii | 0.903 | 0 | 0.8 | 0 | 0.927 | 0 | 0.881 | 13.3 |
| Strophanthus gratus | 0.894 | 0 | 0.911 | 0 | 0.933 | 0 | 0.929 | 0 |
| Strophanthus preussii | 0.819 | 0 | 0.753 | 0 | 0.902 | 0 | 0.898 | 0 |
| Styrax japonicus | 0.958 | 0 | 0.865 | 0 | 0.968 | 0 | 0.953 | 0 |
| Syagrus romanzoffiana | 0.867 | 0 | 0.848 | 0 | 0.914 | 0 | 0.889 | 0 |
| Symplocos paniculata | 0.903 | 0 | 0.806 | 0 | 0.95 | 0 | 0.935 | 0 |
| Syngonium angustatum | 0.905 | 0 | 0.857 | 0 | 0.921 | 0 | 0.92 | 0 |
| Syngonium podophyllum | 0.827 | 0 | 0.849 | 0 | 0.899 | 0 | 0.894 | 0 |
| Syringa reticulata | 0.703 | 0 | 0.849 | 0 | 0.906 | 0 | 0.881 | 0 |
| Syzygium paniculatum | 0.863 | 0 | 0.839 | 0 | 0.931 | 0 | 0.924 | 0 |
| Tabebuia aurea | 0.742 | 0 | 0.742 | 0 | 0.88 | 0 | 0.869 | 0 |
| Tabernaemontana divaricata | 0.8 | 0 | 0.787 | 0 | 0.817 | 0 | 0.803 | 0 |
| Tacca leontopetaloides | 0.874 | 0 | 0.872 | 0 | 0.911 | 0 | 0.903 | 0 |
| Tagetes lucida | 0.911 | 0 | 0.915 | 0 | 0.913 | 0 | 0.903 | 0 |
| Tagetes tenuifolia | 0.858 | 0 | 0.921 | 0 | 0.883 | 0 | 0.857 | 0 |
| Tamarindus indica | 0.77 | 0 | 0.793 | 0 | 0.843 | 0 | 0.833 | 0 |
| Tamarix aphylla | 0.804 | 0 | 0.807 | 0 | 0.828 | 0 | 0.812 | 0 |
| Tamarix chinensis | 0.748 | 0 | 0.732 | 0 | 0.822 | 0 | 0.806 | 0 |
| Tanacetum coccineum | 0.835 | 0 | 0.809 | 0 | 0.939 | 0 | 0.933 | 0 |
| Tecoma stans | 0.671 | 0 | 0.7 | 0 | 0.838 | 0 | 0.794 | 0 |
| Tephrosia candida | 0.766 | 0 | 0.712 | 0 | 0.839 | 0 | 0.839 | 0 |
| Tephrosia grandiflora | 0.937 | 0 | 0.89 | 0 | 0.95 | 0 | 0.938 | 0 |
| Tephrosia purpurea | 0.756 | 0 | 0.763 | 0 | 0.82 | 0 | 0.809 | 0 |
| Tephrosia vogelii | 0.806 | 0 | 0.786 | 0 | 0.854 | 0 | 0.85 | 0 |
| Terminalia catappa | 0.806 | 0 | 0.806 | 0 | 0.859 | 0 | 0.853 | 0 |
| Thalia geniculata | 0.797 | 0 | 0.8 | 0 | 0.878 | 0 | 0.87 | 0 |
| Theobroma cacao | 0.868 | 0 | 0.865 | 0 | 0.907 | 0 | 0.907 | 0 |
| Thunbergia alata | 0.744 | 0 | 0.764 | 0 | 0.862 | 0 | 0.854 | 0 |
| Thunbergia erecta | 0.778 | 0 | 0.745 | 0 | 0.786 | 0 | 0.796 | 0 |
| Thunbergia fragrans | 0.844 | 0 | 0.816 | 0 | 0.843 | 0 | 0.834 | 0 |
| Tigridia pavonia | 0.889 | 0 | 0.839 | 0 | 0.881 | 0 | 0.872 | 0 |
| Tillandsia stricta | 0.927 | 0 | 0.915 | 0 | 0.923 | 0 | 0.92 | 0 |
| Tillandsia usneoides | 0.718 | 0 | 0.75 | 0 | 0.843 | 0 | 0.831 | 40 |
| Tithonia rotundifolia | 0.767 | 0 | 0.748 | 0 | 0.835 | 0 | 0.822 | 0 |
| Toona ciliata | 0.867 | 0 | 0.856 | 0 | 0.908 | 0 | 0.901 | 0 |
| Trachelospermum asiaticum | 0.947 | 0 | 0.942 | 0 | 0.957 | 0 | 0.958 | 40 |
| Trachelospermum jasminoides | 0.768 | 0 | 0.762 | 0 | 0.86 | 0 | 0.845 | 0 |
| Tradescantia spathacea | 0.749 | 0 | 0.749 | 0 | 0.84 | 0 | 0.831 | 0 |
| Triadica sebifera | 0.893 | 0 | 0.847 | 0 | 0.928 | 0 | 0.915 | 0 |
| Trichocentrum carthagenense | 0.881 | 0 | 0.87 | 0 | 0.902 | 0 | 0.891 | 0 |
| Trichosanthes cucumerina | 0.875 | 0 | 0.903 | 0 | 0.924 | 0 | 0.914 | 0 |
| Trichosanthes kirilowii | 0.803 | 0 | 0.801 | 0 | 0.948 | 0 | 0.957 | 0 |
| Tripsacum dactyloides | 0.777 | 0 | 0.81 | 0 | 0.894 | 0 | 0.848 | 0 |
| Tulbaghia violacea | 0.888 | 0 | 0.852 | 0 | 0.906 | 0 | 0.897 | 0 |
| Ullucus tuberosus | 0.935 | 0 | 0.901 | 0 | 0.954 | 0 | 0.954 | 0 |
| Ulmus parvifolia | 0.781 | 0 | 0.718 | 0 | 0.827 | 0 | 0.814 | 0 |
| Ursinia anthemoides | 0.988 | 0 | 0.944 | 0 | 0.988 | 0 | 0.976 | 0 |
| Ursinia speciosa | 0.961 | 0 | 0.946 | 0 | 0.971 | 0 | 0.962 | 0 |
| Utricularia livida | 0.862 | 0 | 0.869 | 0 | 0.918 | 0 | 0.886 | 63.3 |
| Vallisneria americana | 0.751 | 0 | 0.838 | 0 | 0.876 | 0 | 0.871 | 0 |
| Vallisneria nana | 0.591 | 0 | 0.824 | 0 | 0.832 | 0 | 0.816 | 20 |
| Verbena stricta | 0.944 | 0 | 0.943 | 0 | 0.98 | 0 | 0.975 | 0 |
| Vernicia fordii | 0.904 | 0 | 0.877 | 0 | 0.906 | 0 | 0.896 | 0 |
| Veronica americana | 0.748 | 0 | 0.798 | 0 | 0.895 | 0 | 0.833 | 0 |
| Viburnum dilatatum | 0.927 | 0 | 0.911 | 0 | 0.965 | 0 | 0.962 | 0 |
| Viburnum plicatum | 0.918 | 0 | 0.88 | 0 | 0.951 | 0 | 0.949 | 0 |
| Viburnum sieboldii | 0.933 | 0 | 0.918 | 0 | 0.959 | 0 | 0.938 | 0 |
| Vigna caracalla | 0.797 | 0 | 0.714 | 0 | 0.857 | 0 | 0.837 | 0 |
| Viola hederacea | 0.981 | 0 | 0.976 | 0 | 0.986 | 0 | 0.973 | 0 |
| Viola sororia | 0.835 | 0 | 0.845 | 0 | 0.924 | 0 | 0.886 | 0 |
| Vitex negundo | 0.772 | 0 | 0.782 | 0 | 0.85 | 0 | 0.835 | 0 |
| Vitis coignetiae | 0.823 | 0 | 0.848 | 0 | 0.854 | 3.3 | 0.846 | 3.3 |
| Washingtonia robusta | 0.768 | 0 | 0.856 | 0 | 0.872 | 0 | 0.847 | 0 |
| Weigela floribunda | 0.841 | 0 | 0.852 | 0 | 0.953 | 0 | 0.931 | 0 |
| Westringia fruticosa | 0.97 | 0 | 0.931 | 0 | 0.947 | 0 | 0.94 | 0 |
| Wisteria floribunda | 0.922 | 0 | 0.91 | 0 | 0.919 | 0 | 0.916 | 0 |
| Xanthosoma sagittifolium | 0.839 | 0 | 0.783 | 0 | 0.872 | 0 | 0.86 | 0 |
| Ximenia americana | 0.73 | 0 | 0.745 | 0 | 0.839 | 0 | 0.824 | 0 |
| Zapoteca portoricensis | 0.818 | 0 | 0.8 | 0 | 0.861 | 0 | 0.859 | 0 |
| Zelkova serrata | 0.912 | 0 | 0.874 | 0 | 0.916 | 0 | 0.905 | 0 |
| Zingiber officinale | 0.871 | 0 | 0.688 | 0 | 0.827 | 0 | 0.803 | 0 |
| Zingiber zerumbet | 0.821 | 0 | 0.738 | 0 | 0.83 | 0 | 0.84 | 0 |
| Zinnia angustifolia | 0.727 | 0 | 0.761 | 0 | 0.876 | 0 | 0.864 | 0 |
| Zinnia elegans | 0.465 | 66.7 | 0.624 | 0 | 0.718 | 3.3 | 0.697 | 3.3 |
| Zinnia peruviana | 0.827 | 0 | 0.845 | 0 | 0.901 | 0 | 0.888 | 0 |
| Ziziphus mauritiana | 0.713 | 0 | 0.732 | 0 | 0.796 | 0 | 0.799 | 0 |
| Ziziphus spina-christi | 0.837 | 0 | 0.776 | 0 | 0.857 | 0 | 0.843 | 0 |
