# Supplementary material for: Climate change will increase the naturalization risk from garden plants in Europe
Source: Glob Ecol Biogeogr. 2016 Aug 25;26(1):43–53. doi: 10.1111/geb.12512 (PMC5216452; doi:10.1111/geb.12512)
Supplement: Supplementary file 5 — Appendix S5 Naturalization risk maps calculated according to three different weighting schemes. [file GEB-26-43-s005.docx]

*Global Ecology and Biogeography*

**Supporting Information**

**Climate change will increase the naturalization risk from garden plants in Europe**

Iwona Dullinger, Johannes Wessely, Oliver Bossdorf, Wayne Dawson, Franz Essl, Andreas Gattringer, Günther Klonner, Holger Kreft, Michael Kuttner, Dietmar Moser, Jan Pergl, Petr Pyšek, Wilfried Thuiller, Mark van Kleunen, Patrick Weigelt, Marten Winter, Stefan Dullinger

**Appendix S5.** Naturalization risk maps calculated according to three different weighting schemes.

Naturalization risk maps calculated by combining climatic and land cover suitability for 783 ornamental species currently not naturalized in, but somewhere outside of Europe. Land cover suitability was quantified by weighting CORINE land cover types according to the estimated area available for ornamental plant cultivation according to three different weighting schemes (A: (a)-(d), B: (e)-(h), C: (i)-(l), see Appendix S3 for details). Climatic suitability is quantified by projections of species distribution models under current climate ((a), (e), (i)) and three scenarios of climate warming: mild scenario (RCP2.6: (b), (f), (j)), intermediate scenario (RCP4.5: (c), (g), (k)) and strong scenario (RCP8.5: (d), (h), (l)).


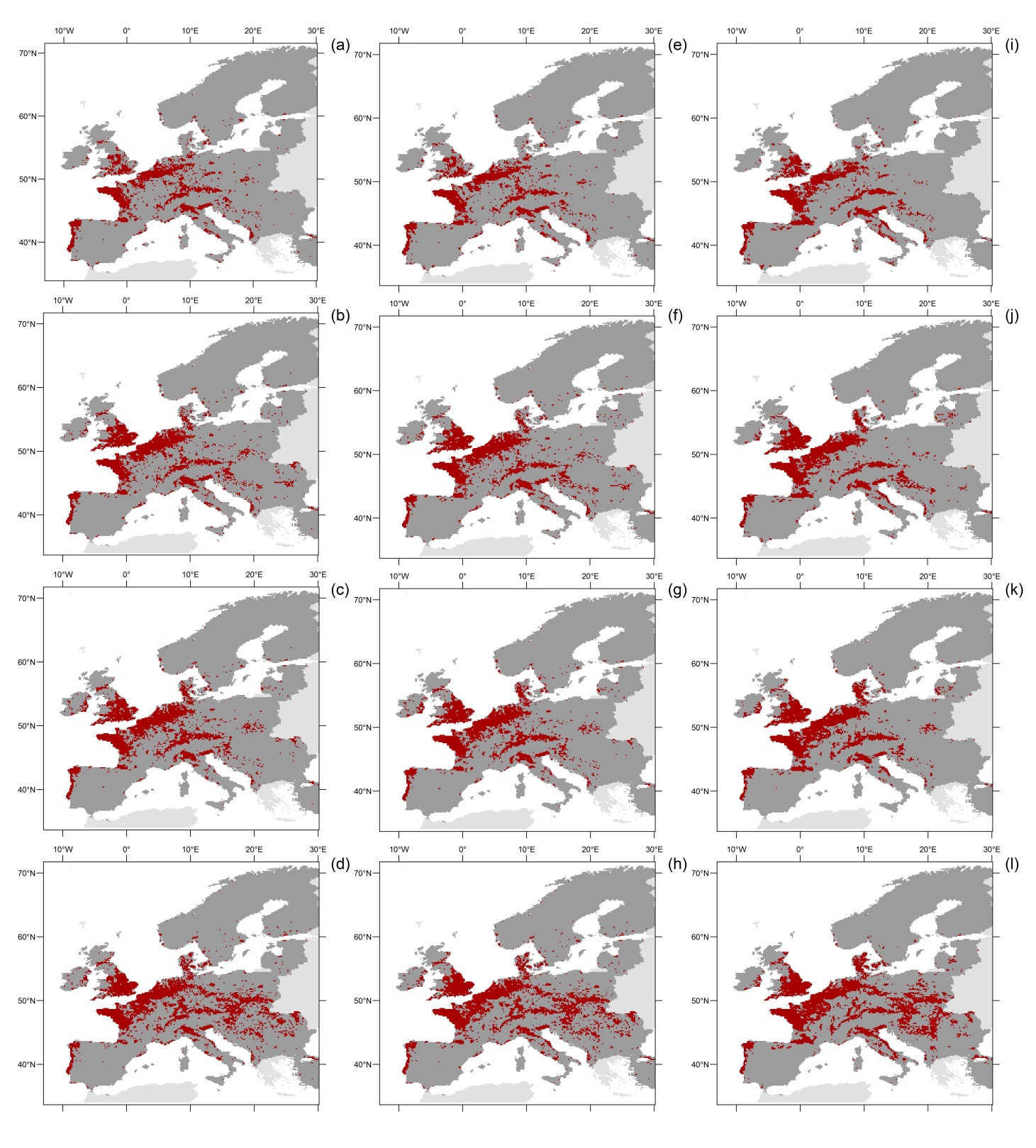


REFERENCES

EEA (2000) *CORINE land cover technical guide – Addendum 2000. Technical report No 40*. http://www.eea.europa.eu/publications/tech40add
