# Supplementary material for: Climate change will increase the naturalization risk from garden plants in Europe
Source: Glob Ecol Biogeogr. 2016 Aug 25;26(1):43–53. doi: 10.1111/geb.12512 (PMC5216452; doi:10.1111/geb.12512)
Supplement: Supplementary file 6 — Appendix S6 Species predicted gain or loss of area under climate change. [file GEB-26-43-s006.docx]

*Global Ecology and Biogeography*

**Supporting Information**

**Climate change will increase the naturalization risk from garden plants in Europe**

Iwona Dullinger, Johannes Wessely, Oliver Bossdorf, Wayne Dawson, Franz Essl, Andreas Gattringer, Günther Klonner, Holger Kreft, Michael Kuttner, Dietmar Moser, Jan Pergl, Petr Pyšek, Wilfried Thuiller, Mark van Kleunen, Patrick Weigelt, Marten Winter, Stefan Dullinger

**Appendix S6.** Species predicted gain or loss of area under climate change.

The proportion of species predicted to gain or lose > 1600 cells (~ 5 % of the study area) of climatically suitable area under three different climate scenarios as compared to current climatic conditions. Orange, red and dark red bars represent ‘winners’, and light blue, blue and dark blue bars represent ‘losers’ under the RCP2.6, RCP4.5, RCP8.5 scenarios, respectively. BORE: boreal, T-AR: temperate-arid, NEMO: nemoral (= temperate), LAUR: laurophyllous, MEDI: Mediterranean, ST-A: subtropical-arid, ST-W: subtropical seasonally dry, TROP: tropical.
